# Combined Computational and Experimental Studies of Anabasine Encapsulation by Beta-Cyclodextrin

**DOI:** 10.3390/plants11172283

**Published:** 2022-08-31

**Authors:** Zeinolla Muldakhmetov, Serik Fazylov, Oral Nurkenov, Arstan Gazaliev, Akmaral Sarsenbekova, Irina Pustolaikina, Zhangeldy Nurmaganbetov, Olzhas Seilkhanov, Aisha A. Alsfouk, Eslam B. Elkaeed, Ibrahim H. Eissa, Ahmed M. Metwaly

**Affiliations:** 1 Institute of Organic Synthesis and Coal Chemistry, Karaganda 100012, Kazakhstan; 2 Department of Physical and Analytical Chemistry, Chemistry Faculty, Karagandy University of the Name of Academician E.A. Buketov, Karaganda 100024, Kazakhstan; 3 Laboratory of NMR-Spectroscopy, Sh. Ualikhanov Kokshetau University, Kokshetau 020000, Kazakhstan; 4 Department of Pharmaceutical Sciences, College of Pharmacy, Princess Nourah Bint Abdulrahman University, P.O. Box 84428, Riyadh 11671, Saudi Arabia; 5 Department of Pharmaceutical Sciences, College of Pharmacy, AlMaarefa University, Riyadh 13713, Saudi Arabia; 6 Pharmaceutical Medicinal Chemistry & Drug Design Department, Faculty of Pharmacy (Boys), Al-Azhar University, Cairo 11884, Egypt; 7 Pharmacognosy and Medicinal Plants Department, Faculty of Pharmacy (Boys), Al-Azhar University, Cairo 11884, Egypt; 8 Biopharmaceutical Product Research Department, Genetic Engineering and Biotechnology Research Institute, City of Scientific Research and Technological Applications, Alexandria 21934, Egypt

**Keywords:** inclusion complex, clathrate, β-cyclodextrin, anabasine, substrate, thermal decomposition of β-cyclodextrin

## Abstract

The encapsulation of the famous alkaloid, anabasine, with β-CD was studied to obtain a more stable and bioavailable inclusion complex. Various in silico and experimental studies of the obtained β-CD-anabasine complex are presented. Firstly, molecular docking studies were conducted against the α, β, and γ cyclodextrins to explore which subclass is the best for encapsulation. The obtained results that pointed at β-cyclodextrin were further confirmed by five MD simulations and MM-PBSA studies. Experimentally, the spectral properties of the anabasine β-cyclodextrin complex were determined by FT-IR, ^1^H, and ^13^C-NMR spectroscopic methods. Additionally, the surface morphology of the anabasine β-cyclodextrin was investigated using a scanning electron microscope. Furthermore, the outputs of the thermographic measurements utilizing a differential scanning calorimeter were displayed. The activation energy of the reaction of thermo-oxidative destruction of the clathrate complex was calculated, and the kinetic parameters of the thermal destruction processes were decided using the Freeman–Carroll, Sharpe–Wentworth, Achar, and Coates–Redfern methods. The kinetic parameters of the thermal decomposition of the anabasine β-cyclodextrin were in agreement and verified the reliability of the obtained results. The obtained computational, spectral, morphological, and thermogravimetric results verified the successful formation of the anabasine β-cyclodextrin complex.

## 1. Introduction

Compounds of natural products have been the most generous source of leads and treatments [1,2]. Anabasine is the well-known alkaloid contained in the herb *Anabasis aphylla* L. [3,4]. Anabasine in small doses excites the central nervous system, enhances breathing, and increases blood pressure. Anabasine exhibited a treating potential against schizophrenia with an ability to enhance the cognitive symptoms through its agonist effect against the α_7_-nicotinic acetylcholine receptors that caused the antagonizing of the MK-801-elicited mouse popping performance at a safe dose that did not induce clinic seizures [5]. Anabasine was an ingredient in three remedies in the Chinese Pharmacopoeia (1977) against rheumatoid arthritis, neuralgia, and sciatica [6]. Additionally, anabasine hydrochloride is used to facilitate the withdrawal from smoking [7,8]. Unfortunately, under industrial conditions, anabasine (colorless liquid) is easily turned into a brown color by the action of atmospheric oxygen and ultraviolet rays [9,10]. Therefore, it is essential to obtain water-soluble encapsulated forms to protect and improve the biofunctional properties of that interesting alkaloid.

One of the most promising and intensively developing areas of modern supramolecular chemistry is the nano-sized products and the inclusion of natural compounds with cyclodextrins (CD) [11,12,13,14]. Cyclodextrins (CD) are a group of cyclic oligosaccharides with an external hydrophilic shell and an internal hydrophobic cavity. CDs are produced through the biochemical starch transformation. The CD group includes α-, β-, and γ-CD, macro rings which consist of six, seven, and eight glucopyranose moieties, respectively [15].

These macro rings can be described as truncated cone-shaped structures with a hydrophobic cavity that binds with the hydrophobic «guest» molecules to form a supramolecular–nanostructured inclusion complex. This guest–host complex provides significant changes in the physical and chemical properties of the guest compound. These changes include a higher degree of stability against oxygen or light [16], more solubility, and the masking of unpleasant odors and tastes [17]. In this regard, it was of interest to study the process of encapsulation of the anabasine molecule with α-, β-, and γ-CD to obtain a supramolecular inclusion complex to create its new dosage form.

The presented work aims to conduct an examination of the encapsulation process of anabasine by beta-cyclodextrin through several in silico (docking, MD simulations, and MM-PBSA) studies and experimental (spectral, surface morphology, and thermographic) techniques.

## 2. Results and Discussion

### 2.1. In Silico Studies

#### 2.1.1. Molecular Docking of Anabasine against α-, β-, γ-cyclodextrins

The molecular docking technique is a successful approach to expect the complexation of cyclodextrins and target compounds [18]. It has been successfully utilized in several studies before [19]. The molecular docking was utilized here to explore the binding pattern of anabasine with the α, β, and γ CD. The goal of this investigation is to determine the most appropriate type of cyclodextrin to be complexed with anabasine. The α, β, and γ CD cavities are O-shaped, consisting of 6, 7, and 8 glucose units, respectively.

Firstly, the binding of the anabasine alkaloid against α CD exerted a binding energy of −17.71 kcal/mol. The obtained binding energy was not the best comparing that of β and γ CD.

**Figure 1:**
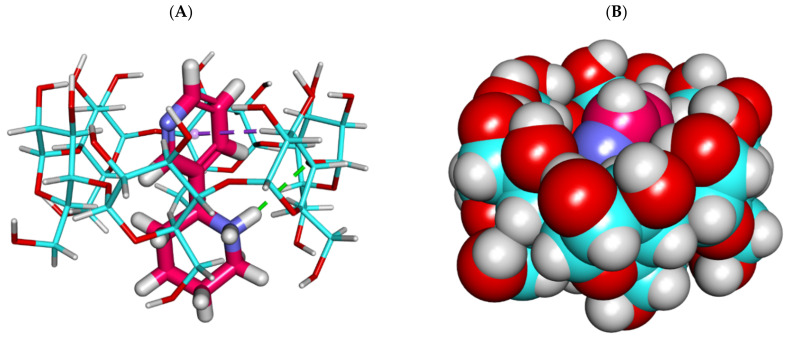
The best-ranked docking poses of anabasine with α CD, front view, as sticks (**A**), and CPK (**B**).

**Figure 2:**
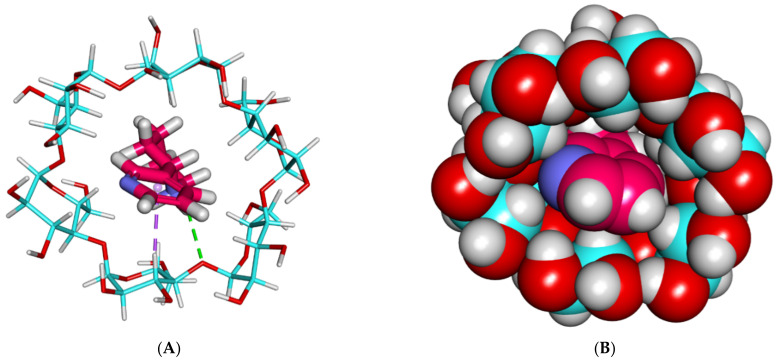
The best-ranked docking poses of anabasine with α CD, top view, as sticks (**A**), and CPK (**B**).

The proposed binding mode of the anabasine alkaloid against α CD formed one hydrogen bond with the OH of glucose subunits through its piperidine moiety. In addition, it formed one hydrophobic interaction through its pyridine moiety (Figure 1 and Figure 2). Additionally, a clear deformation occurs due to the insufficient size of the cyclodextrin cavity to accommodate the entire anabasine molecule.

Secondly, the anabasine alkaloid exhibited a much better binding mode with β and γ CD, where anabasine afforded a high binding score with the β of −21.48 kcal/mol, respectively.

**Figure 3:**
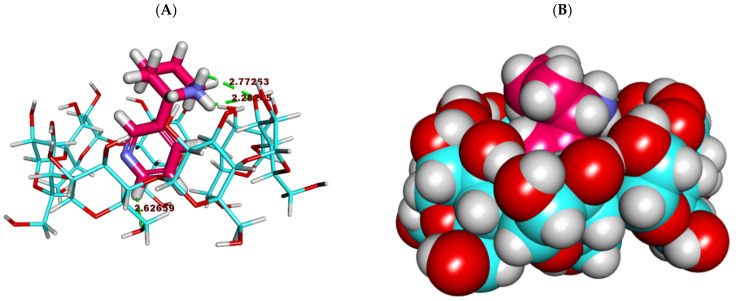
The best-ranked docking poses of anabasine with β CD, front view, as sticks (**A**), and CPK (**B**).

**Figure 4:**
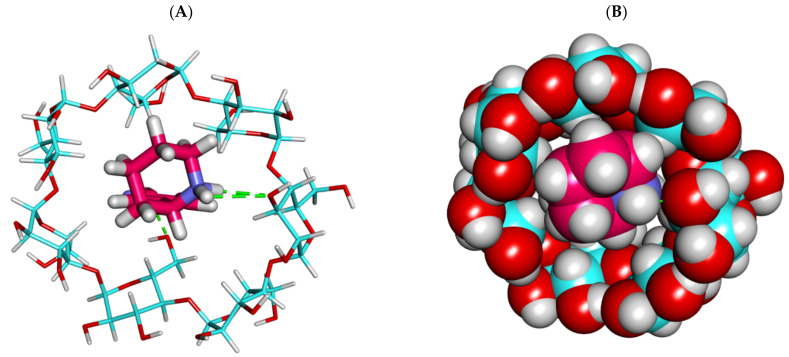
The best-ranked docking poses of anabasine with β CD, top view, as sticks (**A**), and CPK (**B**).

The binding mode of the anabasine alkaloid against β CD formed several vital interactions. The piperidine ring of the anabasine alkaloid formed two hydrogen bonds with the various OHs of the glucose moieties. The pyridine ring of the anabasine alkaloid formed one hydrogen bond with the OH of the glucose subunits (Figure 3 and Figure 4).

**Figure 5:**
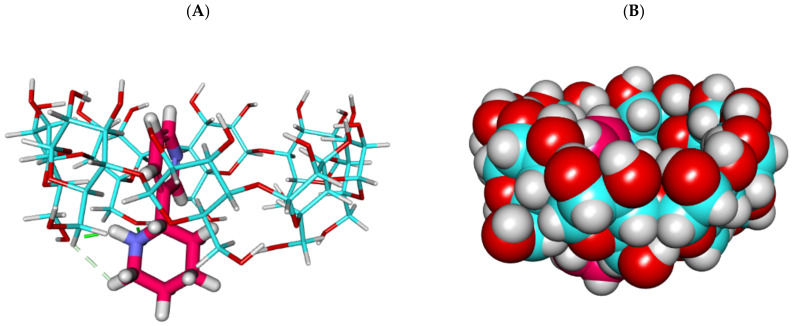
The best-ranked docking poses of anabasine with γ CD, front view, as sticks (**A**), and CPK (**B**).

**Figure 6:**
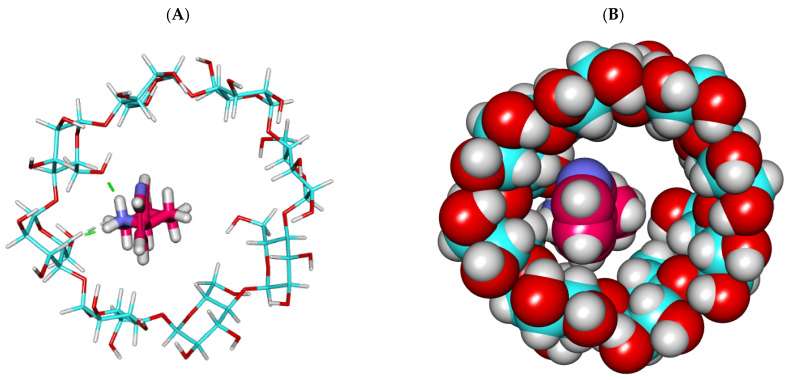
The best-ranked docking poses of anabasine with γ CD, top view, as sticks (**A**), and CPK (**B**).

Finally, anabasine exhibited a good binding mode with γ CD where the anabasine alkaloid produced a high binding energy of −21.29 kcal/mol. The binding mode of the anabasine alkaloid against γ CD formed two hydrogen bonds with the various OHs of the glucose moieties through its piperidine moiety (Figure 5 and Figure 6). Although the binding score of anabasine and γ CD is very near to that of anabasine and β CD, the mode showed that the cavity of γ CD was not occupied properly, with anabasine leaving a big space that may be filled with solvents or other impurities.

These findings indicated that the binding of anabasine with β CD is more advantageous.

#### 2.1.2. Molecular Dynamics (MD) Simulations

Studies based on molecular docking can clearly depict how a ligand binds to a target. A docking study has the disadvantage that it describes the target’s interaction as a rigid unit. Due to this, docking experiments do not account for the calculation of conformational and or energetic changes after a compound binds to a target. Contrarily, the MD simulation experiments can uncover and analyze the structural changes and behavior of both the target and ligand with atomic resolution. Due to its precision, the MD simulation experiment can correctly explore the exact binding mode of a ligand to a target on the levels of conformation and energy [20].

**Figure 7:**
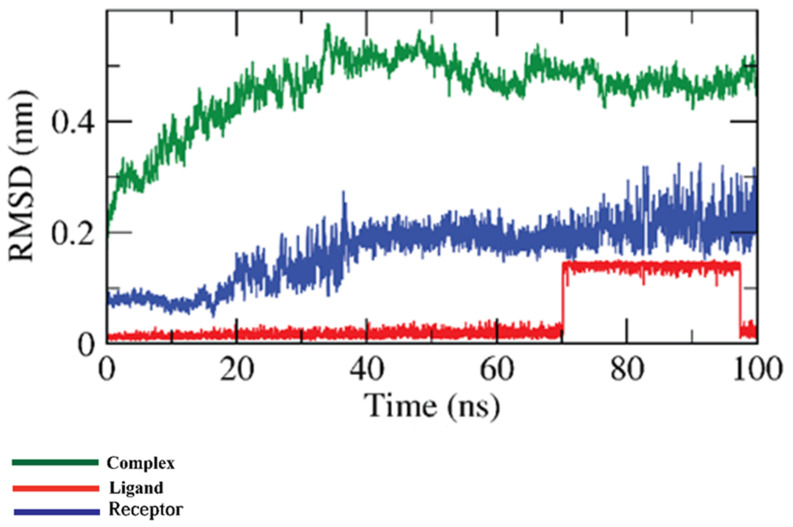
RMSD values of β CD-anabasine complex.

The conformational changes that happened in the β CD-anabasine complex were explored by various MD simulation studies. At first, the RMSD was computed to understand the stability and integration of the β CD-anabasine complex upon the apo and binding states over 100 ns. The β CD-anabasine complex was stable till 70 ns~ and then showed a minor fluctuation. Fortunately, the anabasine-β CD complex became stable again before 100 ns (Figure 7).

**Figure 8:**
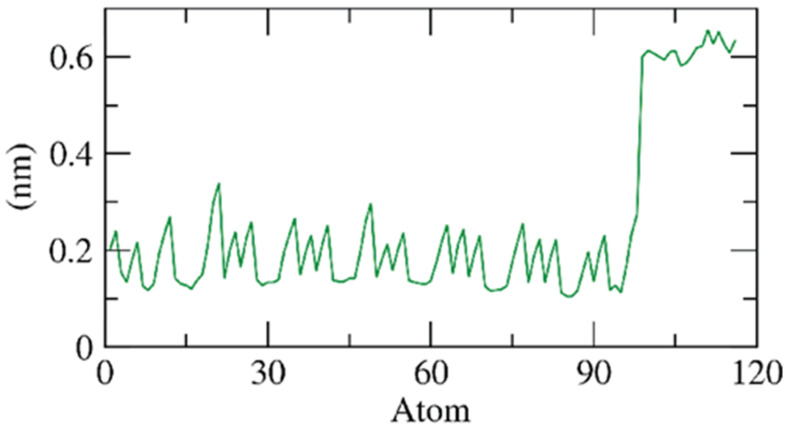
RMSF of β CD-anabasine complex.

Secondly, the flexibility of the β CD-anabasine complex was calculated over the resolution of atoms in terms of the RMSF. The computation of the RMSF determines the region of the β CD that has fluctuated because of the binding process. Figure 8 demonstrates that the anabasine’s binding makes the β CD slightly flexible in 100–120 atom (ligand) areas.

**Figure 9:**
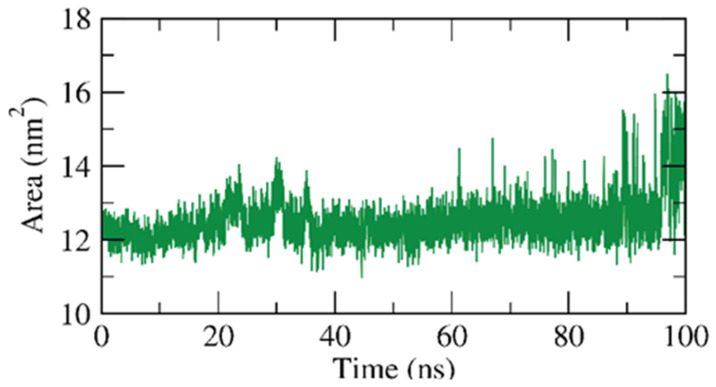
SASA of β CD-anabasine complex.

Additionally, the interactions between the β CD-anabasine complex and the enclosing solvents were estimated by the solvent accessible surface area (SASA) over the 100 ns of the experiment. The SASA values of any complex are a strong indicator of the conformational changes that eventuated due to the interaction of its components. Fascinatingly, as shown in Figure 9, the β CD featured an integration in the surface area showing a relatively stable SASA value at the end of the simulations comparing with the starting time.

**Figure 10:**
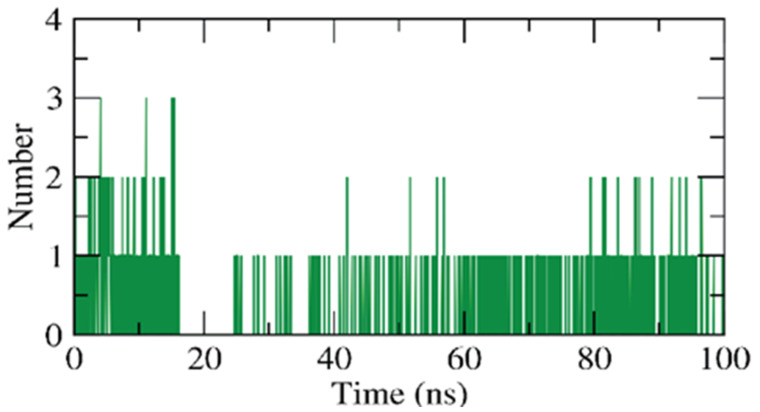
H-bonding of β CD-anabasine complex.

The hydrogen bonding between the β CD-anabasine complex was explored as it is a very essential factor to stabilize the complex. Figure 10 demonstrates that the highest hydrogen bond number of the β CD formed most of the time is up to two bonds with anabasine, while it reached three bonds at different periods of the simulations.

**Figure 11:**
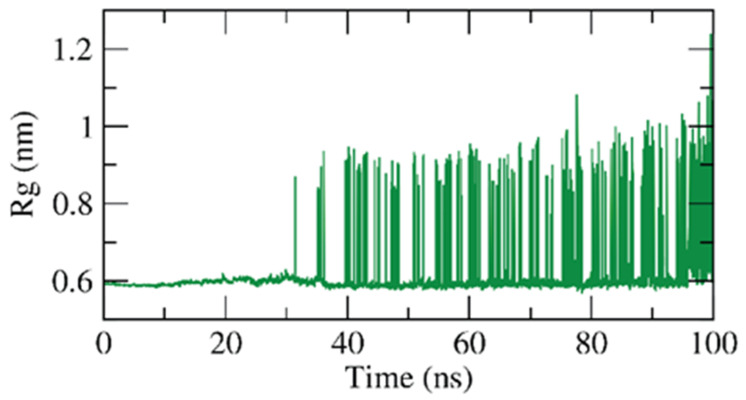
Rg of the β CD-anabasine complex.

The radius of gyration, (Rg), is a constant parameter to determine the stability of the target based on changes in its volume, and the Rg is the RMSD of a weighted mass unit of atoms from their mass center. It is thought that the Rg indicates 3D changes in the target besides its compactness, and the fluctuation during the simulation time is inversely proportional to the stability of the system, Thus, the Rg of the β CD-anabasine complex was computed to be almost stable for the examined 100 ns with slight fluctuations (0.6–1 nm), as shown in Figure 11, indicating the stability and compactness of the β CD-anabasine complex.

#### 2.1.3. MMPBSA of the β CD-Anabasine Complex

**Figure 12:**
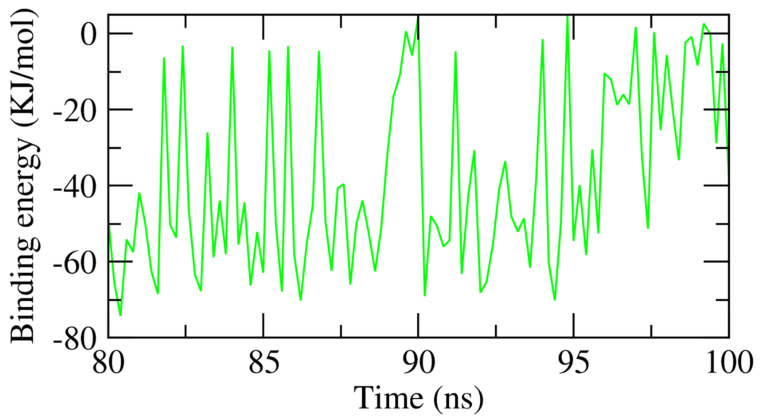
MM-PBSA findings of anabasine-β CD complex.

The accurate binding energy of the β CD-anabasine complex at the final 20 ns of the MD experiment was computed with an interval of 100 ps from the MD trajectories. The MM/PBSA method and the MmPbSaStat.py script were utilized in these calculations to explore the average free binding energy in addition to its standard deviation or error from the obtained files of the g_mmpbsa. As Figure 12 shows, anabasine showed an exact binding energy of −38 kJ/mol with the β CD.

### 2.2. Experimental Studies

#### 2.2.1. Surface Morphology Study of the Anabasine-β-CD Complexes Samples

**Figure 13:**
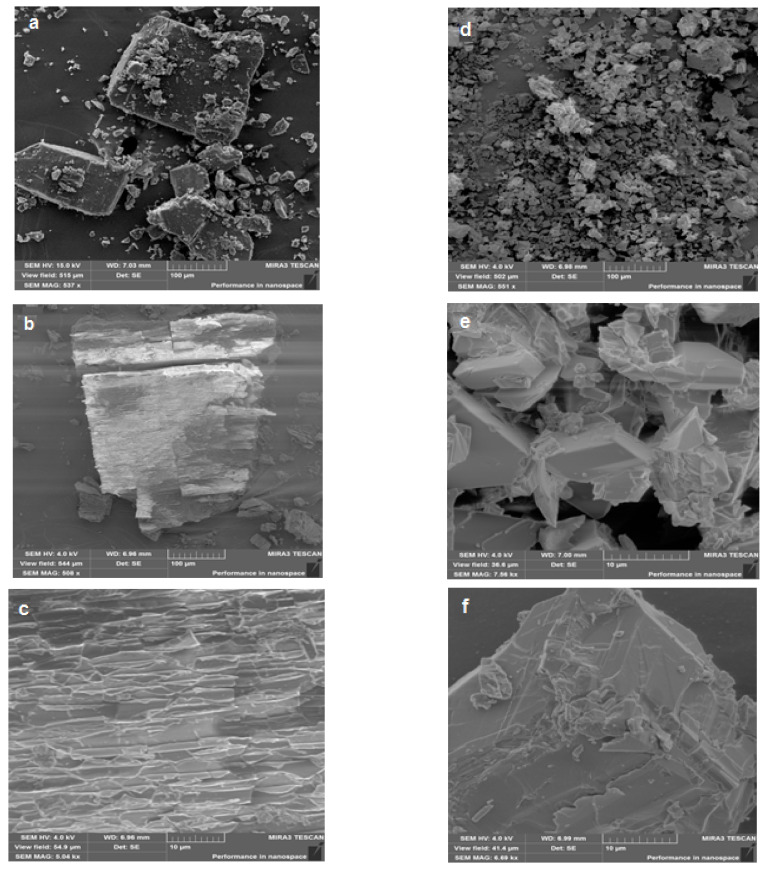
Scanned electron micrographs of β-CD (**a**–**c**) and the anabasine- β CD (1:1) inclusion complex (**d**–**f**) at various magnifications (Tescon Mira3 LMN).

Figure 13 shows the scanned electron micrographs of β-CD (a-c) and the anabasine-β CD (1:1) inclusion complex (d–e) at different magnifications (Tescon Mira3 LMN). The images were obtained at the accelerating voltages of 3 and 7 kV (SEM MAP 537x-8.06 kx). The photos show the structure of β-CD (a and b) and the pattern of the anabasine- β CD clathrate (c and d) at various magnifications. As follows from the analysis of these images, there is a change in the crystal morphology of the samples of the anabasine- β CD (1:1) inclusion complexes. The change in the crystal surface morphology of the anabasine- β CD (1:1) clathrate complexes is important evidence of the formation of the inclusion of the complex.

#### 2.2.2. Study of the Structure of the Obtained Anabasine-β-CD Supramolecular Inclusion Complexes

**Figure 14:**
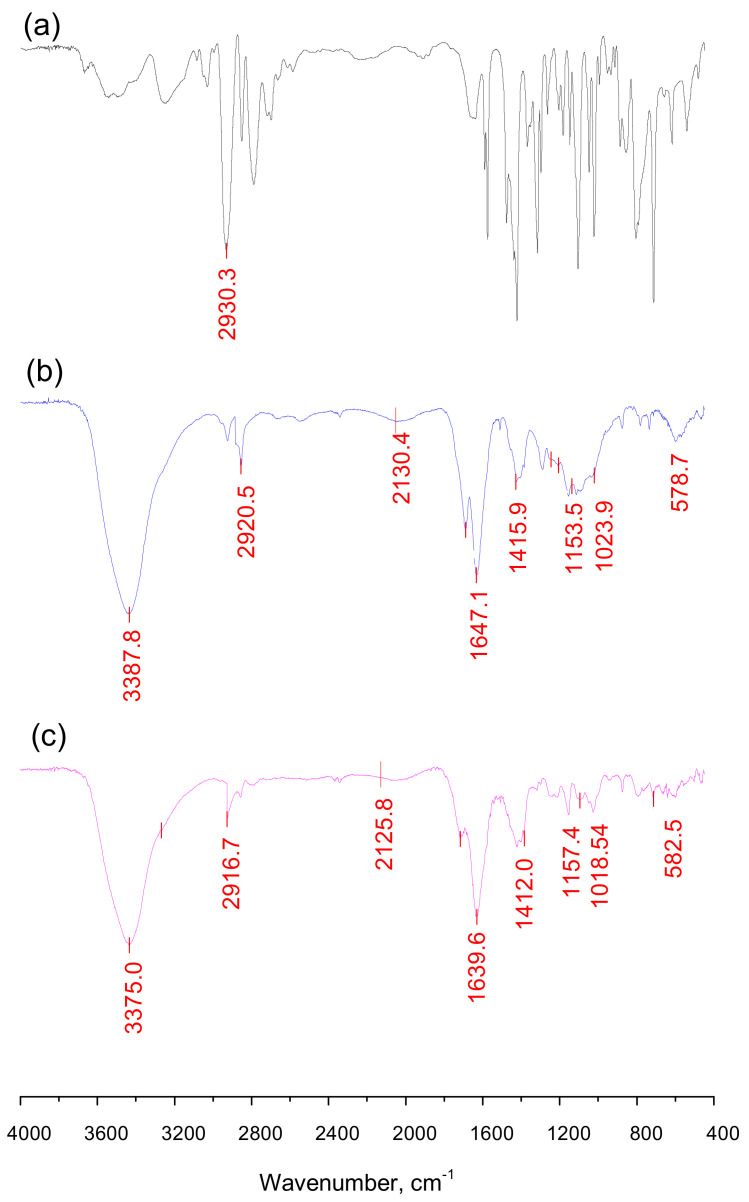
FTIR spectra of anabasine (**a**), β-CD (**b**), and its inclusion complex (**c**) (KBr).

The structures of the obtained anabasine-β CD inclusion complexes were previously confirmed using IR Fourier, NMR ^1^H, and ^13^C spectroscopy and the two-dimensional spectra ROESY (^1^H-^1^H). Figure 14 shows the IR spectra of anabasine (a) and β-CD (b,c) in the single and clathrate form. In the FTIR spectrum of the anabasine alkaloid at 2920–2930 cm^−1^, the peaks of the aromatic CH groups are visible, and the NH group is manifested at 3350–3400 cm^−1^. The vibrations of the carbon skeleton (C-C bonds) of the anabasine and β-CD molecules manifest themselves in the form of intense bands in the region of 1100–1211 cm^−1^. Hydroxyl groups of the β-CD appear at 3387 cm^−1^ in the form of an intense wide band. In the IR spectrum of the clathrate anabasine-β-CD, small shifts in the characteristic absorption bands of the β-CD functional groups were noted, indicating the absence of covalent interactions between the anabasine and the β-CD torus. For example, the valence vibrations of the O-H bonds of the β-CD in the complex are detected as a wide band with a maximum at 3375 cm^–1^, undergoing a shift of 12 cm^−1^. The absorption bands of β-CD at 2924 cm^−1^ undergo similar small displacements, which are characteristic of the valence vibrations of C-H bonds in the methine and methylene groups. The absorption band at 1651 cm^−1^ denotes the deformation vibrations of OH bonds in the SON groups, and those at 1423, 1364, and 1335 cm^−1^ denote the deformation vibrations of the C-H bonds in the CH_2_OH and CHOH groups. Intense absorption bands in the region of 2800–3450 cm^−1^ and 1350–1470 cm^−1^ indicate the saturated nature of the complex. Absorption bands of the C=C and NH groups of anabasine did not appear. This may be because of the masking effect of the intense and very wide β-CD bands in the same range of the wavelength.

The study of supramolecular inclusion complexes of the guest: hosttype between β-CD and different substrates by NMR-^1^H spectroscopy (JNM-ECA Jeol 400) is based on the determination of the difference in the values of the ^1^H and ^13^C chemical shifts in the substrate (anabasine) and the receptor (β-CD) in a single state and as a part of the complexes. The supramolecular interaction of the “guest” and “host” molecules leads to a shift in the NMR signals of their atoms. There is a significant chemical shift for the (H-2, H-3, H-4, H-5, and H-6) protons of the anabasine piperidine ring in comparison with the (H-8, H-10, H-11, and H-12) protons of the anabasine pyridine ring (Table 1).

**Figure 15:**
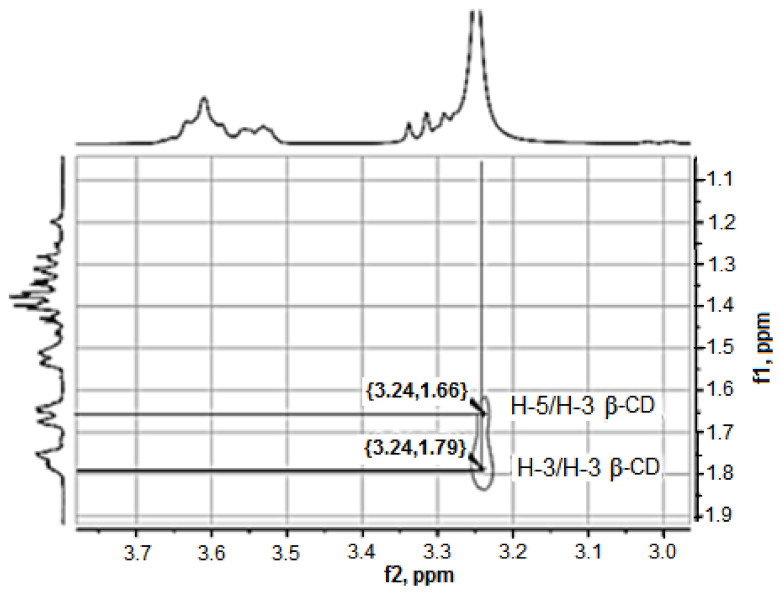
Fragment of the ROESY spectrum of the anabasine-β CD inclusion complex.

The analysis of the data obtained suggests that the greatest participation in the complexation with the “host” molecule occurs with the participation of the piperidine fragment of the “guest” molecule. For the ^1^H cyclodextrin nuclei, the formation of complexes is accompanied by a shift of the signals to the weak field. The largest chemical shifts in the protons are observed for the H-3 (0.132 ppm) and H-5 (0.097 ppm) atoms, which are directed to the inner part of the cyclodextrin cone. Based on these data, it can be concluded that the supramolecular interaction of anabasine with β-CD leads to the formation of an inclusion complex. The formation of a 1:1 supramolecular compound is also confirmed by the comparison of the values of the integral intensities of the anabasine and β-CD protons in the anabasine-β CD inclusion complex. The formation of the anabasine-β CD inclusion complex was also confirmed by the analysis of the 2D ^1^H NMR ROESY spectrum (Figure 15). The cross peaks of the proton–proton interactions of the H-3 and H-5 atoms of β-CD with the protons of the anabasine piperidine fragment were observed in the two-dimensional spectrum.

**Figure 16:**
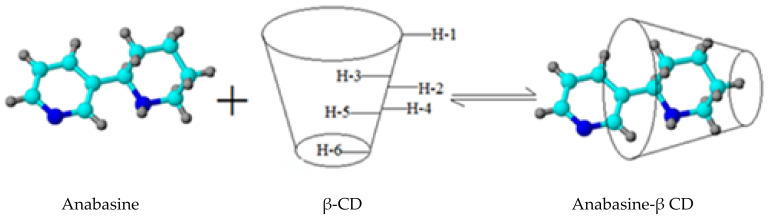
Diagram of the inclusion complex anabasine-β CD formation.

An analysis of the spectral data shows the absence of covalent interactions between anabasine and the β-CD. Nonspecific (dispersion and van der Waals) interactions play a decisive role in the clathrate complex formation. Based on the analysis of the data obtained, it can be assumed that the participation of the piperidine fragment of the anabasine molecule makes a significant contribution to the complexation with the «host» molecule (Figure 16).

#### 2.2.3. Thermal Analysis of Anabasine-β CD Inclusion Complexes Samples by Differential Thermogravimetry and Differential Scanning Calorimetry

**Figure 17:**
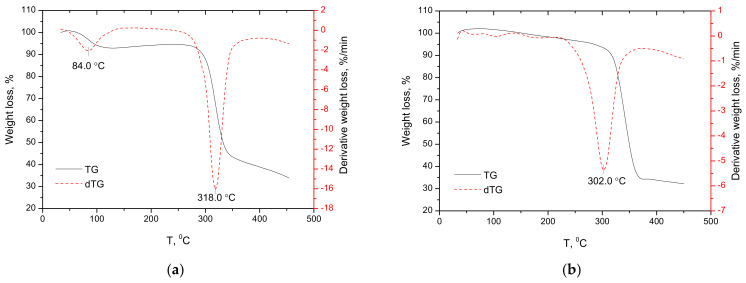

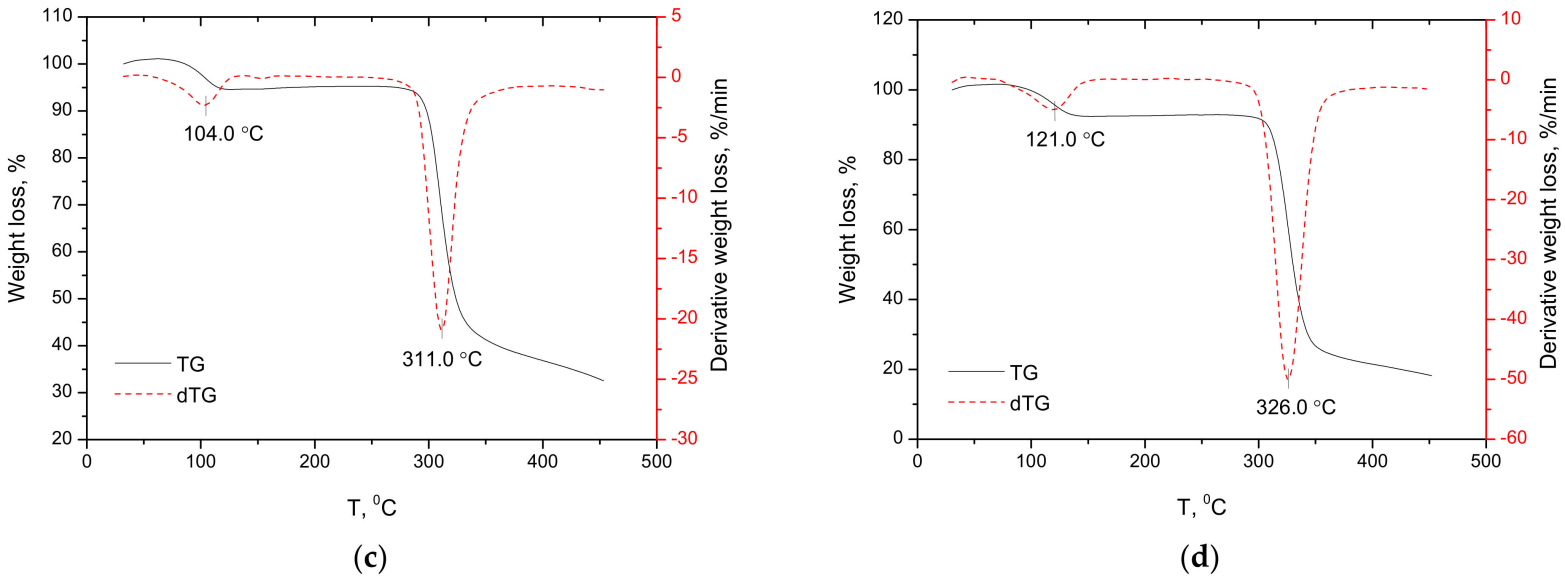
TG/DTG curves of β-CD (**a**) (in nitrogen)), anabasine-β CD 1:1 (**b**); 1:2 (**c**); 1:3 (**d**) complexes at a constant heating rate of 10 degree/min (in air).

The combined employing of several methods to characterize the physical condition of the studied objects gives more reliable results in terms of model stability [21,22]. In this regard, it was interesting to compare the energy and thermodynamic characteristics of the anabasine-β CD clathrate. The thermal stability of anabasine and its clathrates with β-CD in a ratio of 1:1, 1:2, and 1:3 was estimated by differential scanning calorimetry [23,24]. The parameters of the thermal analysis of the decomposition of anabasine-β CD (1:1)–(1:3) are presented on the Tg/DTG curves (Figure 17a–c). An analysis of the TG and DTG curves (Figure 17a–c) showed that the studied anabasine-β CD clathrates contained bound water, like β-CD. The dehydration endothermic peak of the examined samples was in the range of 70–109 °C (Figure 17a–c) [22,23,24].

**Figure 18:**
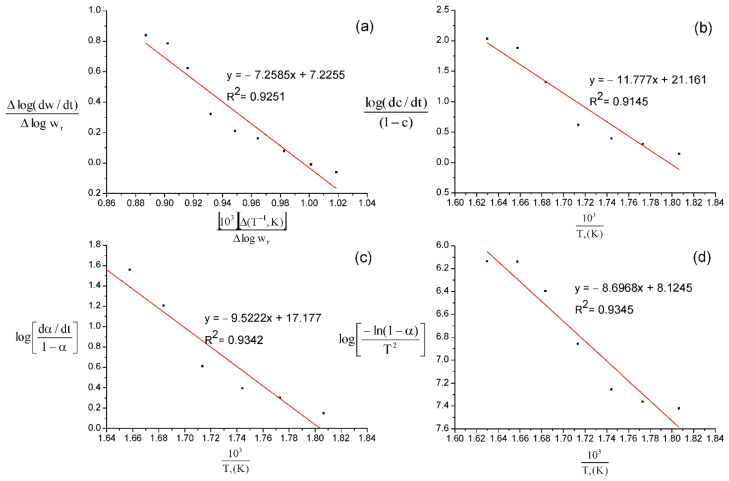
Processing of TGA anabasine-β CD 1:1 data by Freeman–Carroll (**a**), Sharpe–Wentworth (**b**), Ahar (**c**), and Coates–Redfern (**d**) methods.

The mathematical processing of the thermogravimetric curves of anabasine-β CD (1:1), shown in Figure 18, made it possible to determine the activation energy (E) using the Freeman–Carroll (a) [23], Sharpe–Wentworth (b) [24], Achar (c) [25], and Coates–Redfern (d) methods [26]. Similar TGA calculations were performed for the clathrates anabasine-β CD (1:2) and (1:3). The obtained kinetic parameters of the thermal destruction of the clathrates are presented in Table 2. The above models determined graphically the thermodynamic parameters through the process of the thermal decomposition of β-CD and its clathrates with anabasine.

**Freeman–Carroll method:***In this method, the following expression is used*.

$$\frac{\Delta \log\left(\frac{dw}{dt}\right)}{\Delta \log w_{r}}=\frac{-\frac{E_{a}}{2.303R}\cdot \Delta \left(\frac{1}{T}\right)}{\Delta \log w_{r}+n}$$

where $\frac{dw}{dt}=\mathrm{rate}$ of change in mass of β-CD and its clathrates with anabasine with respect to time $w_{r}=w_{c}-w$, where $w_{c}$ is the loss of the mass at the completion of the β-CD and its clathrates reaction or at specific time, and w is the total mass up to time t. T is the temperature, R is the gas constant, and n is the order of reaction. Hence, the graph of $\frac{\Delta \log\left(\frac{dw}{dt}\right)}{\Delta \log w_{r}}$ versus $\frac{\Delta \left(\frac{1}{T}\right)}{\Delta \log w_{r}}$.

Should give the value of the order of reaction n on “Y” axis and the slope m = −E_a_/2.303R. The detailed procedure is clearly laid out for samples as an illustration.

**Sharp–Wentworth method:***In this method, the following expression is used*.

$$\log\frac{dc/dt}{1-c}=\log\alpha /\beta =E_{a}/2.303\;RT$$

where β is the linear heating rate. The graph of $\log\frac{dc/dt}{1-c}$ versus 1/T on “X” axis has been plotted. The graph is a straight line with E_a_ as slope and A as intercept on “Y” axis. The linear relationship confirms that the assumed order n = 1 is correct.

## 3. Experimental

### 3.1. Geometric Parameters of α-, β-, γ-cyclodextrins and Anabasine

Carried out by the MM^+^ molecular mechanics’ method using the HyperChem 8.0 program. (thoroughly described in the Supplementary Materials).

### 3.2. Molecular Docking

Docking studies were carried out using Discovery studio software 4.0, 2016 (Vélizy-Villacoublay, France) [27] (thoroughly y described in the Supplementary Materials).

### 3.3. MD Simulations

CHARMM-GUI was used to prepare the system. Moreover, CHARMM36 force field and NAMD 2.13 package and the TIP3P explicit solvation model were used [28,29,30] (thoroughly described in the supplementary materials).

### 3.4. Complex Preparation

Thoroughly described in the Supplementary Materials.

### 3.5. Spectroscopic Measurements

Thoroughly described in the Supplementary Materials.

### 3.6. Surface Morphology

Studied using a scanning electron microscope (SEM) and thoroughly described in the Supplementary Materials.

### 3.7. Thermal Analysis

Carried out by differential thermogravimetry (DTG) and scanning calorimetry (DSC) (thoroughly described in the Supplementary Materials).

## 4. Conclusions

The computational and physicochemical studies of the encapsulation of anabasine with β-cyclodextrin were performed. The molecular docking studies recommended β cyclodextrins as the best option for encapsulation. The MD simulation studies confirmed the excellent binding of the complex. The spectral properties of the anabasine-β cyclodextrin complex were characterized by the SEM, FT-IR, and NMR ^1^H and ^13^C spectroscopy data. The surface morphology of the anabasine-β cyclodextrin complex was explained employing the scanning electron microscope. The activation energy of the reaction of the thermo-oxidative destruction of the clathrate complex is calculated and the kinetic parameters of the thermal destruction processes were determined utilizing the Freeman–Carroll, Sharpe–Wentworth, Achar, and Coates–Redfern methods. The obtained kinetic parameters confirmed the reliability of the complexation.

## Figures and Tables

**Table 1:** Chemical shifts of ^1^H and ^13^C of anabasine and β-CD in the free state and as part of the complex.

Carbon Atom Number	Group	The Value of δ_0_ in the Free State, ppm		The Value of δ in the Composition of the Complex, ppm		The Value of δ in the Composition of the Complex, ∆δ = δ − δ_0_, ppm	
	CH_x_	δ(^1^H)	δ(^13^C)	δ(^1^H)	δ(^13^C)	∆δ(^1^H)	∆δ(^13^C)
Anabasine							
2	-CH_ax_	2.610	47.203	2.610	47.163	0	−0.04
	-CH_eq_	2.991		2.991		0	
3	-CH_2_-	1.882	25.973	1.750	25.952	−0.132	−0.021
4	-CH_2_-	1.452	25.510	1.442	25.480	−0.010	−0.030
5	-CH_2_-	1.471	35.245	1.374	35.212	−0.097	−0.033
6	>CH-	3.542	59.272	3.556	59.335	0.014	−0.06
7	>C=	–	141.602	–	141.571	–	−0.031
8	-CH=N	8.500	148.263	8.490	148.285	−0.010	0.022
10	-CH=N	8.380	148.620	8.380	148.62	0	0
11	-CH=	7.263	123.660	7.274	123.702	0.011	0.042
12	-CH=	7.680	134.522	7.680	134.665	0	0.143
β-CD							
1	>CH-	4.773	102.432	4.796	102.667	0.023	0.235
2	>CH-	3.270	72.875	3.303	72.965	0.033	0.091
3	>CH-	3.49	73.545	3.615	73.685	0.123	0.140
4	>CH-	3.302	82.005	3.345	82.168	0.042	0.163
5	>CH-	3.450	72.525	3.560	72.658	0.110	0.133
6	-CH_2_-	3.572	60.402	3.635	60.576	0.063	0.174

**Table 2:** Kinetic parameters of thermal destruction were obtained by processing the data of TGA clathrates anabasine-β CD (1:1, 1:2, 1:3) with a constant heating rate of 10 deg/min in an air medium according to the Freeman–Carroll, Sharp–Wentworth, Ahar, and Coates–Redfern methods.

	Method	Freeman–Carroll		Sharp–Wentworth		Ahar		Coates–Redfern	
Sample		E, kJ/mol	n	E, kJ/mol	A×10^9^, min^−1^	E, kJ/mol	A×10^7^, min^−1^	E, kJ/mol	A×10^3^, min^−1^
β-CD *		548.71	1.40	712.24	1.27	600.84	1.10	458.70	1.32
Anabasine-β CD 1:1		138.80	1.90	225.20	1.55	182.09	2.88	166.30	3.38
Anabasine-β CD 1:2		328.83	2.81	356.06	4.13	336.70	6.13	281.36	2.40
Anabasine-β CD 1:3		217.25	2.19	312.34	8.06	249.60	4.73	234.92	4.19

*—in the atmosphere of nitrogen.

## Data Availability

Data are available.

## Supplementary Materials

Thoroughly described methods and spectral data of anabasine-β cyclodextrin complex can be downloaded at: https://www.mdpi.com/article/10.3390/plants11172283/s1.

## References

[1] Metwaly A.M., Ghoneim M.M., Eissa I.H., Elsehemy I.A., Mostafa A.E., Hegazy M.M., Afifi W.M., Dou D. (2021). Traditional ancient Egyptian medicine: A review. Saudi J. Biol. Sci..

[2] Han X., Yang Y., Metwaly A.M., Xue Y., Shi Y., Dou D. (2019). The Chinese herbal formulae (Yitangkang) exerts an antidiabetic effect through the regulation of substance metabolism and energy metabolism in type 2 diabetic rats. J. Ethnopharmacol..

[3] Conroy M.S. (2004). Russian-American pharmaceutical relations, 1900–1945. Pharm. Hist..

[4] Sadykov A., Tumur B. (1960). The alkaloids *of Anabasis aphylla* var. iljinii. Dokl. Akad. Nauk Uzb. SSR.

[5] Mastropaolo J., Rosse R.B., Deutsch S.I. (2004). Anabasine, a selective nicotinic acetylcholine receptor agonist, antagonizes MK-801-elicited mouse popping behavior, an animal model of schizophrenia. Behav. Brain Res..

[6] Gong N.-B., Du L.-D., Lu Y. (2018). Anabasine. Natural Small Molecule Drugs from Plants.

[7] Trofimov B.A., Andriyankova L.V., Tlegenov R.T., Mal’kina A.G., Afonin A.V., Il’icheva L.N., Nikitina L.P. (2005). Reaction of anabasine with 3-(1-hydroxycyclohexyl)-2-propynenitrile: A new route to functionalised anabasine alkaloids. Mendeleev Commun..

[8] Babaev B.N., Dalimov D.N., Tiliabaev Z., Tylegenov R.T. (2010). Synthesis, structure and biological properties of phosphorylated anabazine derivatives. Chem. Plant Raw Mater..

[9] Gessner G., Larrañaga M.D., Lewis Sr R.J., Lewis R.A. (1997). Hawley’s Condensed Chemical Dictionary.

[10] Haag H. (1933). A contribution to the pharmacology of anabasine. J. Pharmacol. Exp. Ther..

[11] Kurkov S.V., Loftsson T. (2013). Cyclodextrins. Int. J. Pharm..

[12] Astray G., Gonzalez-Barreiro C., Mejuto J.C., Rial-Otero R., Simal-Gandara J. (2009). A review on the use of cyclodextrins in foods. Food Hydrocoll..

[13] Larsen K.L. (2002). Large cyclodextrins. J. Incl. Phenom. Macrocycl. Chem..

[14] Loftsson T., Másson M., Brewster M.E. (2004). Self-association of cyclodextrins and cyclodextrin complexes. J. Pharm. Sci..

[15] Crini G. (2014). A history of cyclodextrins. Chem. Rev..

[16] Del Valle E.M. (2004). Cyclodextrins and their uses: A review. Process Biochem..

[17] Marques H.M.C. (2010). A review on cyclodextrin encapsulation of essential oils and volatiles. Flavour Fragr. J..

[18] Iskineyeva A., Fazylov S., Bakirova R., Sarsenbekova A., Pustolaikina I., Seilkhanov O., Alsfouk A.A., Elkaeed E.B., Eissa I.H., Metwaly A.M. (2022). Combined In Silico and Experimental Investigations of Resveratrol Encapsulation by Beta-Cyclodextrin. Plants.

[19] Palli V., Leonis G., Zoupanou N., Georgiou N., Chountoulesi M., Naziris N., Tzeli D., Demetzos C., Valsami G., Marousis K.D.J.M. (2022). Losartan Interactions with 2-Hydroxypropyl-β-CD. Molecules.

[20] Hansson T., Oostenbrink C., van Gunsteren W. (2002). Molecular dynamics simulations. Curr. Opin. Struct. Biol..

[21] Serra R., Sempere J., Nomen R. (1998). A new method for the kinetic study of thermoanalytical data:: The non-parametric kinetics method. Thermochim. Acta.

[22] Burkeev M., Fazylov S., Bakirova R., Iskineyeva A., Sarsenbekova A., Tazhbaev E., Davrenbekov S. (2021). Thermal decomposition of β-cyclodextrin and its inclusion complex with vitamin E. Mendeleev Commun..

[23] Coats A.W., Redfern J. (1964). Kinetic parameters from thermogravimetric data. Nature.

[24] Freeman E.S., Carroll B. (1958). The application of thermoanalytical techniques to reaction kinetics: The thermogravimetric evaluation of the kinetics of the decomposition of calcium oxalate monohydrate. J. Phys. Chem..

[25] Sharp J.H., Wentworth S.A. (1969). Kinetic analysis of thermogravimetric data. Anal. Chem..

[26] Achar B.N., Brindley G., Sharp J. Kinetics and mechanism of dehydroxylation processes. III. Applications and limitations of dynamic methods. Proceedings of the International Clay Conference 1966.

[27] Alanazi M.M., Elkady H., Alsaif N.A., Obaidullah A.J., Alkahtani H.M., Alanazi M.M., Alharbi M.A., Eissa I.H., Dahab M.A. (2021). New quinoxaline-based VEGFR-2 inhibitors: Design, synthesis, and antiproliferative evaluation with in silico docking, ADMET, toxicity, and DFT studies. RSC Adv..

[28] Jo S., Kim T., Iyer V.G., Im W. (2008). CHARMM-GUI: A web-based graphical user interface for CHARMM. J. Comput. Chem..

[29] Brooks B.R., Brooks III C.L., Mackerell A.D., Nilsson L., Petrella R.J., Roux B., Won Y., Archontis G., Bartels C., Boresch S. (2009). CHARMM: The biomolecular simulation program. J. Comput. Chem..

[30] Lee J., Cheng X., Swails J.M., Yeom M.S., Eastman P.K., Lemkul J.A., Wei S., Buckner J., Jeong J.C., Qi Y. (2016). CHARMM-GUI Input Generator for NAMD, GROMACS, AMBER, OpenMM, and CHARMM/OpenMM Simulations Using the CHARMM36 Additive Force Field. J. Chem. Theory Comput..

