# Supplementary material for: Combined Computational and Experimental Studies of Anabasine Encapsulation by Beta-Cyclodextrin
Source: Plants (Basel). 2022 Aug 31;11(17):2283. doi: 10.3390/plants11172283 (PMC9460387; doi:10.3390/plants11172283)
Supplement: Supplementary file 1 [file plants-11-02283-s001.zip › plants-1853535-supplementary.pdf]

Supporting data

Zeinolla Muldakhmetov[1], Serik Fazylov[*1,2], Oral Nurkenov[1,2], Arstan Gazaliev[1], Akmaral Sarsenbekova[2], Irina Pustolaikina [2], Zhangeldy Nurmaganbetov[1], Olzhas Seilkhanov [3], Aisha A. Alsfouk[4], Eslam B. Elkaeed[5], Ibrahim H. Eissa[6], Ahmed M. Metwaly *[7,8]

[1] Institute of Organic Synthesis and Coal Chemistry, Karaganda100012, Kazakhstan;

[2] Karagandy University of the name of academician E.A. Buketov 100024, Kazakhstan;

[3] Sh. Ualikhanov Kokshetau University, Kokshetau 020000, Kazakhstan

[4] Department of Pharmaceutical Sciences, College of Pharmacy, Princess Nourah bint Abdulrahman University, P.O. Box 84428, Riyadh 11671, Saudi Arabia

[5]Department of Pharmaceutical Sciences, College of Pharmacy, AlMaarefa University, Riyadh 13713, Saudi Arabia

[6] Pharmaceutical Medicinal Chemistry & Drug Design Department, Faculty of Pharmacy (Boys), Al-Azhar University, Cairo11884, Egypt

[7] Pharmacognosy and Medicinal Plants Department, Faculty of Pharmacy (Boys), Al-Azhar University, Cairo 11884, Egypt

[8]Biopharmaceutical Product Research Department, Genetic Engineering and Biotechnology Research Institute, City of Scientific Research and Technological Applications, Alexandria 21934, Egypt

Method

1. **Molecular Modeling studies**

1.1. Geometric parameters of α-, β-, γ-cyclodextrins and anabasine

Ana:CD was performed by the MM^+ molecular mechanics method using the HyperChem 8.0 program. The chemical structures were taken from the PubChem Substance and Compound database (pubchem.ncbi.nlm.nih.gov). Chemical structure unique identifiers are: CID 444041 for β-cyclodextrin, and CID 205586 for anabasine. The geometry of the complexes was optimized by the MM+ method. All calculations were carried out without taking into account the effect of the solvent (gas phase, vacuum).

1.2.Preparation of the α, β, and γ CD structure

The 3D crystal structures of the α, β, and γ CD were extracted from the cyclo/maltodextrin/ alpha-cyclodextrin complex, beta-cyclodextrin complex, and gamma cyclodextrin complex, respectively. These complexes were retrieved from RCSB Protein Data Bank with PDB IDs of 2ZYM (resolution 1.80 Å), 1BFN (resolution 2.07 Å), and 1p2g (resolution 2.30 Å), respectively. The docking analyses were performed using Discovery Studio 4.0 software to evaluate the free energies and binding mode of Roseofungin molecule with the core site of α, β, and γ CD. The most promising pose were selected depending the increased binding free energy (ΔG).

The 3D crystal structures of α, β, and γ CD were prepared by selecting α, β, and γ CD subunits and removing protein and all water molecules, heteroatoms and co-factors. Moreover, the correction of uncorrected valence atoms and crystallographic disorders were performed using alternate conformations and valence monitor options. Then, the α, β, and γ CD were protonated and its inflexibility was obtained by creating fixed atom constraint. Next, the energy was energy minimized by applying CHARMM (Chemistry at HARvard Macromolecular Mechanics) force fields, and MMFF94 (Merck Molecular force field) force field for charge and partial charge, respectively[4]. The binding sites of the α, β, and γ CD were defined as receptor molecules and prepared for docking.

1.3.Preparation of ligand

The 2D structure of anabazine alkaloid molecule was sketched using ChemBioDraw Ultra 14.0 and saved in MDL-SD file format. Then, the SD file was opened (by Discovery studio 4.0 software) and protonated. Force fields were applied on the molecule to get lowest energy minimum structure via CHARMM and MMFF94 force fields for charge and partial charge, respectively. Then, each of them was prepared for docking by applying ligand preparation protocol.

1.4. Docking simulation

The docking is a technique that can reliably predict the preferred configuration of one molecule relative to another molecule when they are bound to each other to forma stable complex. The evaluation of generated poses was mainly based on the number of interactions they formed with the residues of active site upon binding.

The molecular docking of anabazine alkaloid was performed using CDOCKER protocol which is an implementation of the CDOCKER algorithm. CDOCKER is a grid-based molecular docking method that employs CHARMM-based molecular dynamics (MD) scheme to dock ligands into a receptor binding site . The default values were selected for the CDOCKER protocol. The CDOCKER energy (receptor-ligand interaction energy) of best docked poses was calculated.

1.5.Molecular dynamics simulations

Molecular dynamics simulation of the protein-ligand complexes was performed using GROMACS 2021.1 version and Linux 5.4 package. The GROMOS96 54a7 forcefield was selected as the force field for proteins and the ligand topologies were generated from the PRODRG[2] server. All the complexes were solvated using simple point charge (SPC) water molecules in a rectangular box. To make the simulation system electrically neutral, required number of Na+ and Cl− ions were added while 0.15 mol/L salt concentrations were set in all the systems. Using the steepest descent method, all the solvated systems were subjected to energy minimization for 5000 steps. Afterwards, NVT (constant number of particles, volume, and temperature) series, NPT (constant number of particles, pressure, and temperature) series, and the production run were conducted in the MD simulation. The NVT and the NPT series were conducted at a 300 K temperature and 1 atm pressure for the duration of 300 ps. V-rescale thermostat and Parrinello-Rahman barostat were selected of the performed simulation. Finally, the production run was performed at 300 K for a duration of 100 ns (nanoseconds). Thereafter, a comparative analysis was performed measuring root mean square deviation (RMSD), root mean square fluctuation (RMSF), radius of gyration (Rg), solvent accessible surface area (SASA) and hydrogen bonds to analyze their stability. The Xmgrace program was used to represent the analyses in the form of plots.

2. Complex preparation

The following reagents were used in the experiments:

- anabazine (2-(3-pyridyl)piperidine) (98,5%) («Aldrich»), base was used in the work, which is a light yellowish oil, with 1.01 g/cm^3 density, and 162.23 g/mol molar mass.

- β-cyclodextrin (99.5%) («Fluka»), white crystalline substance, t.p. 280-299°C, soluble in water when heated, well soluble in dimethylsulfoxide and pyridine.

Anabazine inclusion complexes with β-CD were obtained by co-dissolution. Calculated amounts of anabazine and β-CD (mmol) in molar ratios (1:1; 1:2; 1:3) dissolved in a minimum amount of a mixture of solvents water:ethanol (1:1). The solutions were stirred on a magnetic stirrer at 50 ° C for 5 hours. The dropped product was filtered, washed with acetone and dried at a temperature of 30-35°C. Inclusion complexes Ana:β-CD were obtained in the form of a white crystalline powder melting with decomposition at 280-310°C.

3. Spectroscopic measurments

The IR spectra of the inclusion complexes Ana:β-CD were taken on a Cary 600 Series IR Fourier spectrometer manufactured by Agilent Technologies (USA). The ^{1}H and ^{13}C NMR spectra of the obtained clathrates were recorded on a JNM-ECA Jeol 400 spectrometer (frequency 399.78 MHz, DMSO-d_6).

4. Surface morphology

The surface morphology of the samples of the inclusion complexes was studied using a scanning electron microscope (SEM) from Tescon Mira3 LMN (Czech Republic).

5. Thermal analysis

Thermal analysis of samples of Ana:β-CD complexes (12 mg sample *weight*) was carried out by differential thermogravimetry (DTG) and scanning calorimetry (DSC) on a Setaram DTA/DSC calorimeter.

Supplementary

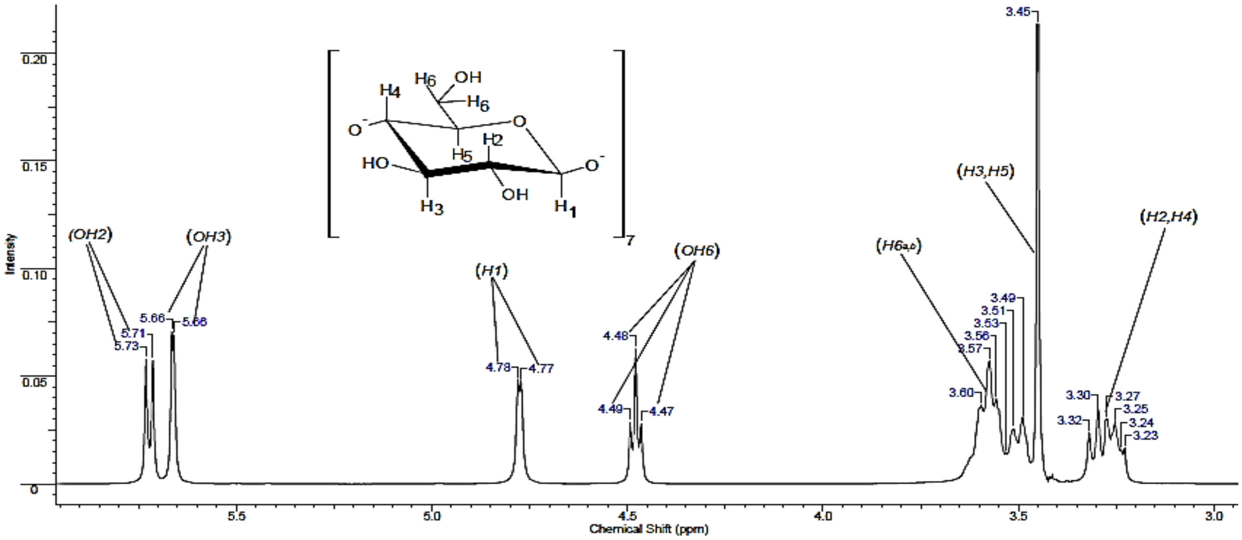

No.	(ppm)	(Hz)	Height	No.	(ppm)	(Hz)	Height
1	3.23	1290.5	0.0166	12	3.57	1428.8	0.0572
2	3.24	1294.1	0.0159	13	3.60	1437.5	0.0366
3	3.25	1300.5	0.0288	14	4.47	1785.0	0.0281
4	3.27	1308.8	0.0303	15	4.48	1790.5	0.0629
5	3.30	1317.9	0.0353	16	4.49	1796.0	0.0288
6	3.32	1327.1	0.0237	17	4.77	1907.8	0.0476
7	3.45	1379.8	1.0000	18	4.78	1911.0	0.0481
8	3.49	1394.9	0.0308	19	5.66	2262.7	0.0693
9	3.51	1404.5	0.0256	20	5.66	2264.5	0.0709
10	3.53	1412.3	0.0142	21	5.71	2284.2	0.0575
11	3.56	1421.9	0.0394	22	5.73	2291.1	0.0580

^{1}H-NMR spectra of free β-CD (spectrometer JNM-ECA Jeol 400 operating at 400 MHz (frequency 399.78) (25°C)

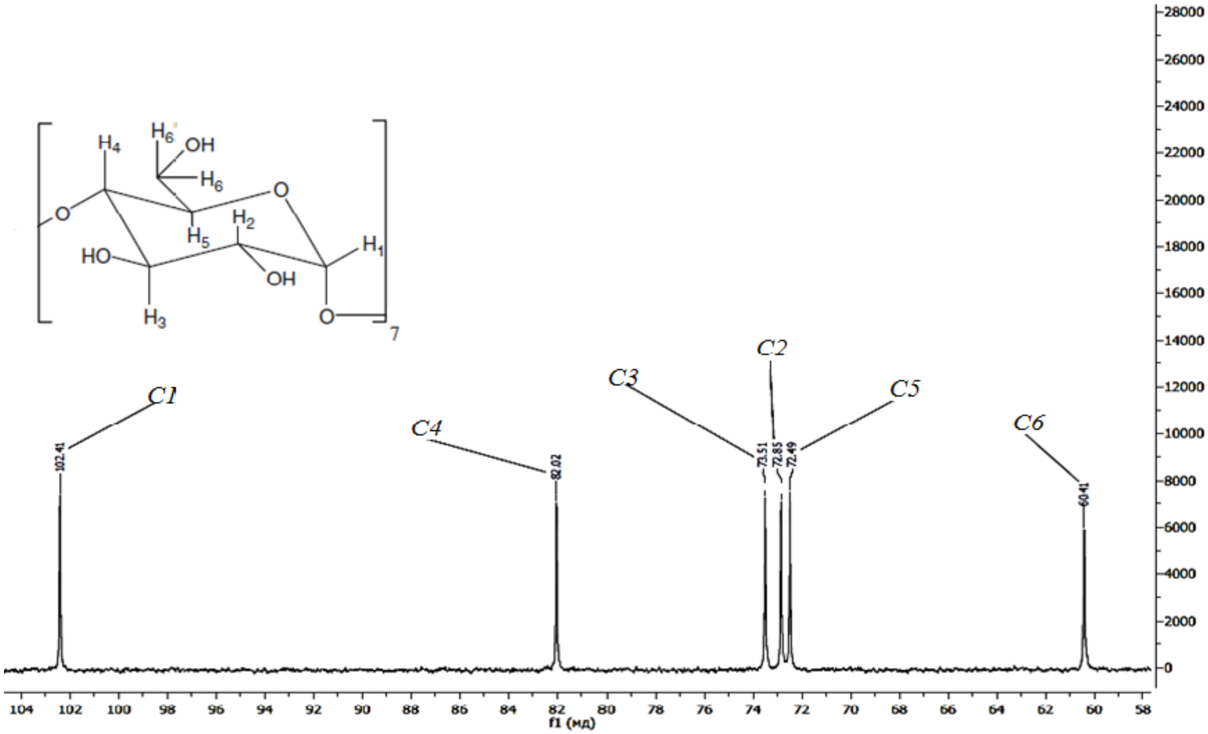

^{13}C NMR spectra of free β-CD

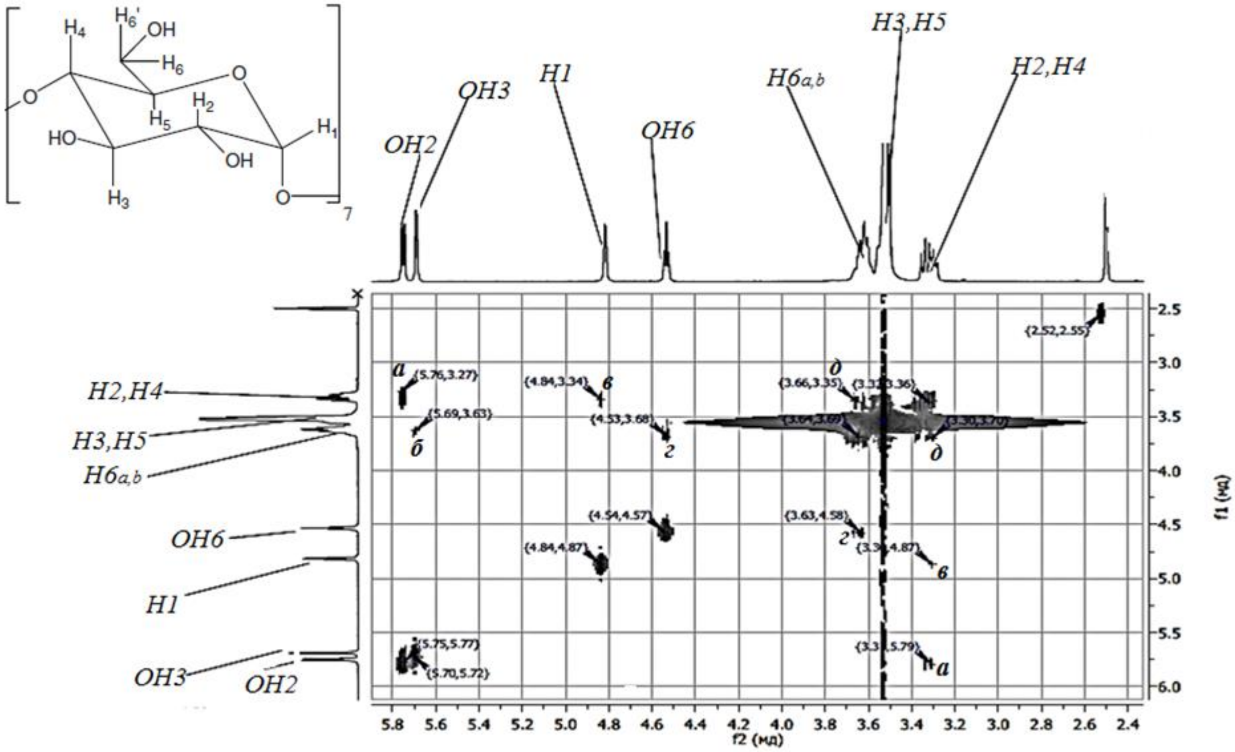

COSY (^{1}H-^{1}H) spectra of β-CD

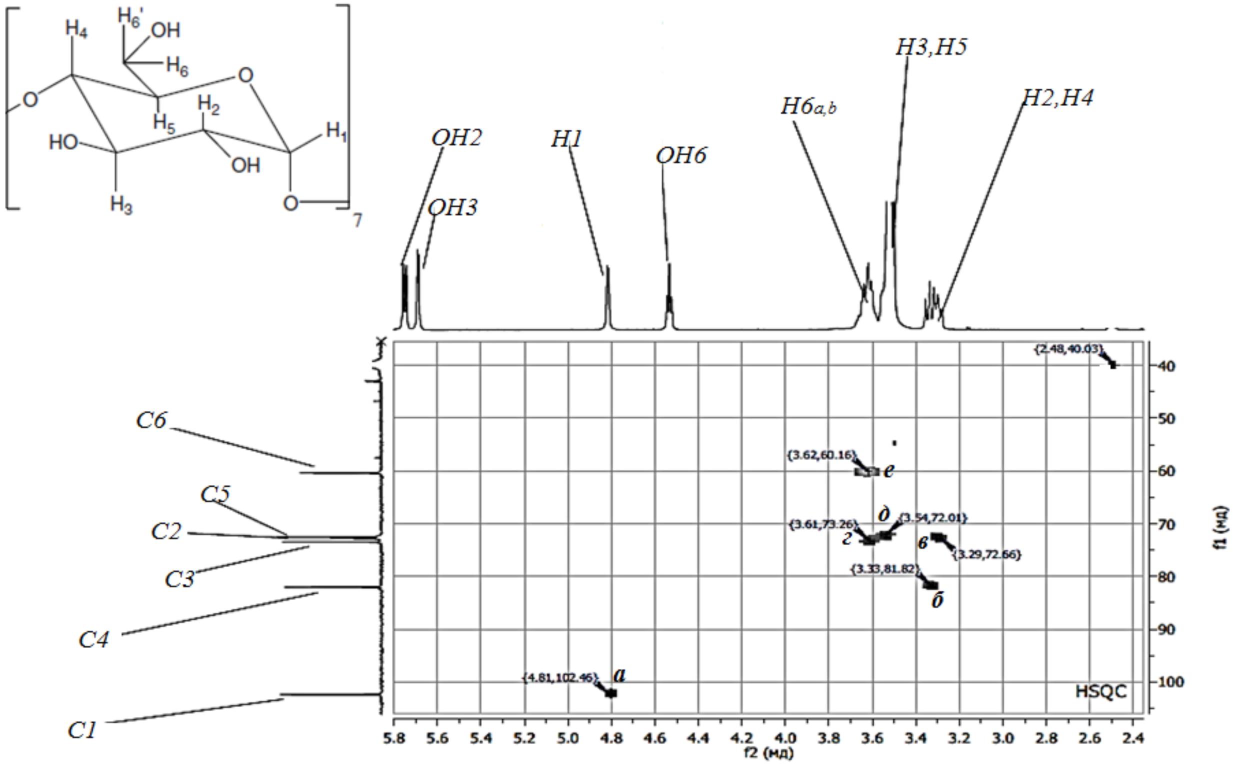

HSQC (^{1}H-^{13}C) spectra of β-CD

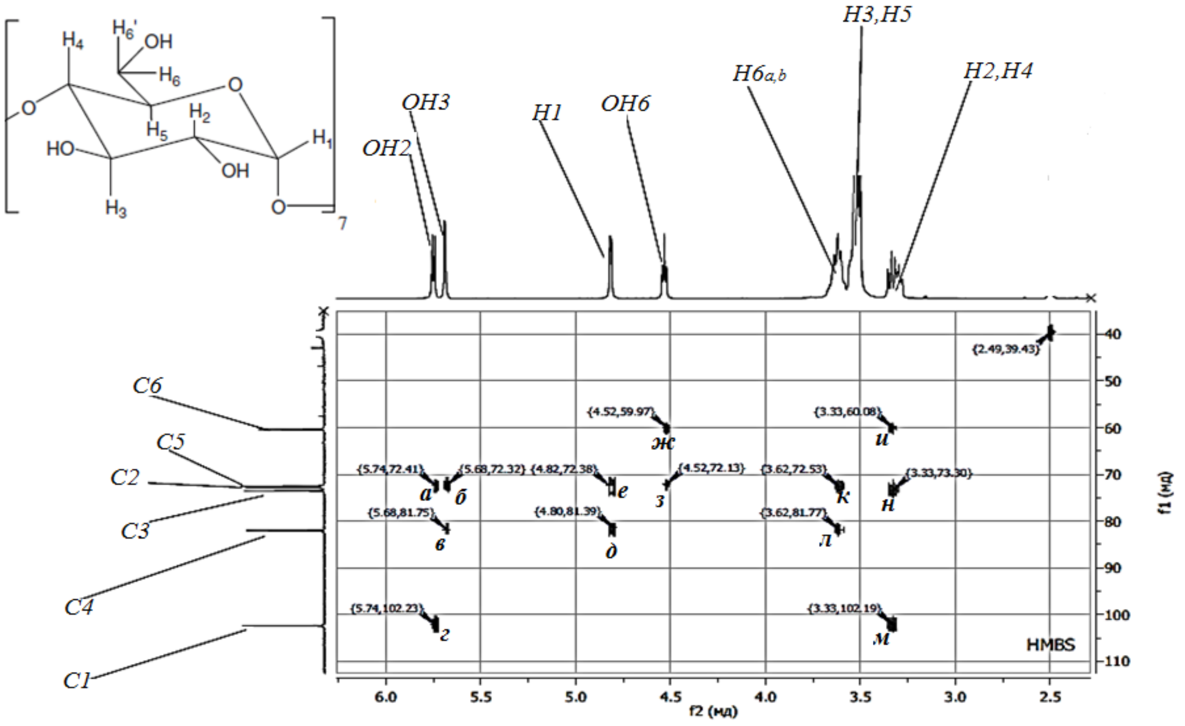

HMBC (^{1}H-^{13}C)

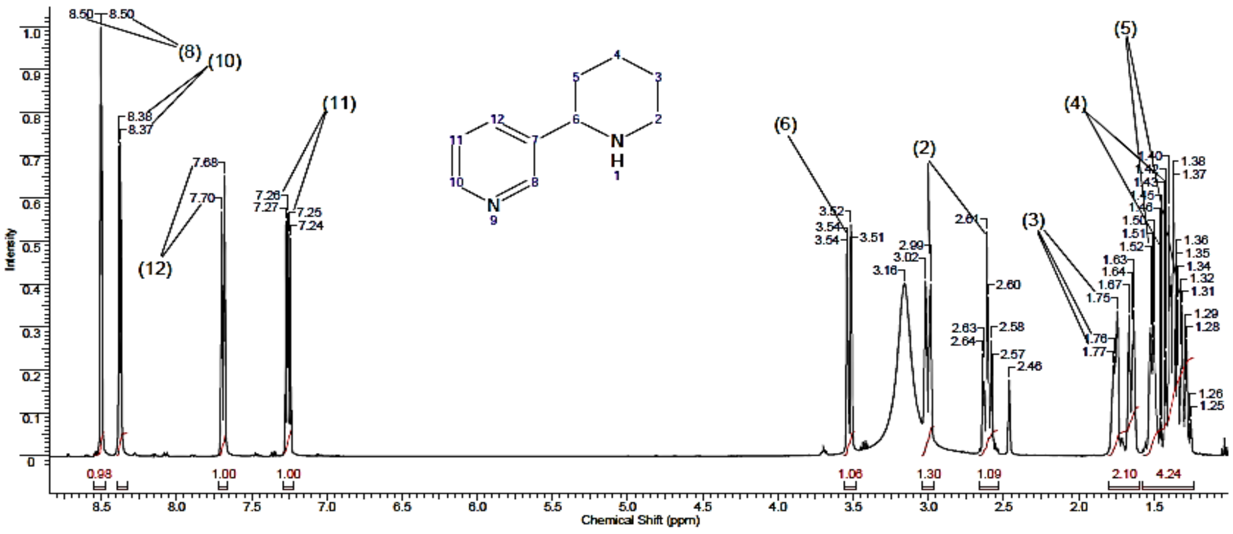

No.	(ppm)	(Hz)	Height	No.	(ppm)	(Hz)	Height	No.	(ppm)	(Hz)	Height	No.	(ppm)	(Hz)	Height
1	1.25	500.5	0.0852	14	1.42	568.3	0.2450	27	2.46	985.0	0.1767	40	3.54	1413.7	0.4358
2	1.26	503.7	0.0756	15	1.43	571.5	0.2238	28	2.47	986.8	0.1279	41	3.54	1416.4	0.4722
3	1.28	512.9	0.2695	16	1.45	580.6	0.0806	29	2.57	1029.4	0.1818	42	7.24	2895.1	0.5074
4	1.29	515.6	0.1714	17	1.46	583.9	0.0895	30	2.58	1032.2	0.2697	43	7.25	2900.2	0.5184
5	1.31	523.9	0.3527	18	1.50	600.3	0.3069	31	2.60	1040.9	0.3696	44	7.26	2902.9	0.5655
6	1.32	527.5	0.2542	19	1.51	602.6	0.2694	32	2.61	1043.6	0.5222	45	7.27	2907.9	0.5479
7	1.34	535.8	0.3644	20	1.52	608.6	0.2995	33	2.63	1052.8	0.2235	46	7.68	3070.0	0.6532
8	1.35	539.0	0.3707	21	1.63	653.5	0.3399	34	2.64	1055.5	0.2376	47	7.70	3077.8	0.5702
9	1.36	542.2	0.1616	22	1.64	656.2	0.3118	35	2.99	1193.4	0.4004	48	8.37	3345.3	0.7138
10	1.37	547.2	0.6262	23	1.67	665.8	0.3291	36	3.02	1207.1	0.4081	49	8.37	3347.1	0.7305
11	1.38	550.4	0.4975	24	1.75	697.9	0.3372	37	3.16	1263.0	0.4014	50	8.38	3350.3	0.7231
12	1.39	556.4	0.5110	25	1.76	704.3	0.2316	38	3.51	1403.1	0.4795	51	8.50	3397.5	0.9992
13	1.40	559.1	0.5819	26	1.77	707.5	0.2118	39	3.52	1405.4	0.5393	52	8.50	3399.8	1.0000

No.	(ppm)	Value	Absolute Value
1	[1.23 .. 1.57]	4.242	7.42340e+1
2	[1.60 .. 1.80]	2.098	3.67186e+1
3	[2.54 .. 2.66]	1.090	1.90737e+1
4	[2.96 .. 3.04]	1.298	2.27171e+1
5	[3.49 .. 3.56]	1.059	1.85290e+1
6	[7.23 .. 7.29]	1.000	1.74983e+1
7	[7.66 .. 7.72]	0.999	1.74765e+1
8	[8.33 .. 8.39]	0.968	1.69450e+1
9	[8.48 .. 8.56]	0.984	1.72214e+1

^{1}H NMR spectra of anabazine (spectrometer JNM-ECA Jeol 400 operating at 400 MHz (frequency 399.78) (25°C), DMSO-d_6)

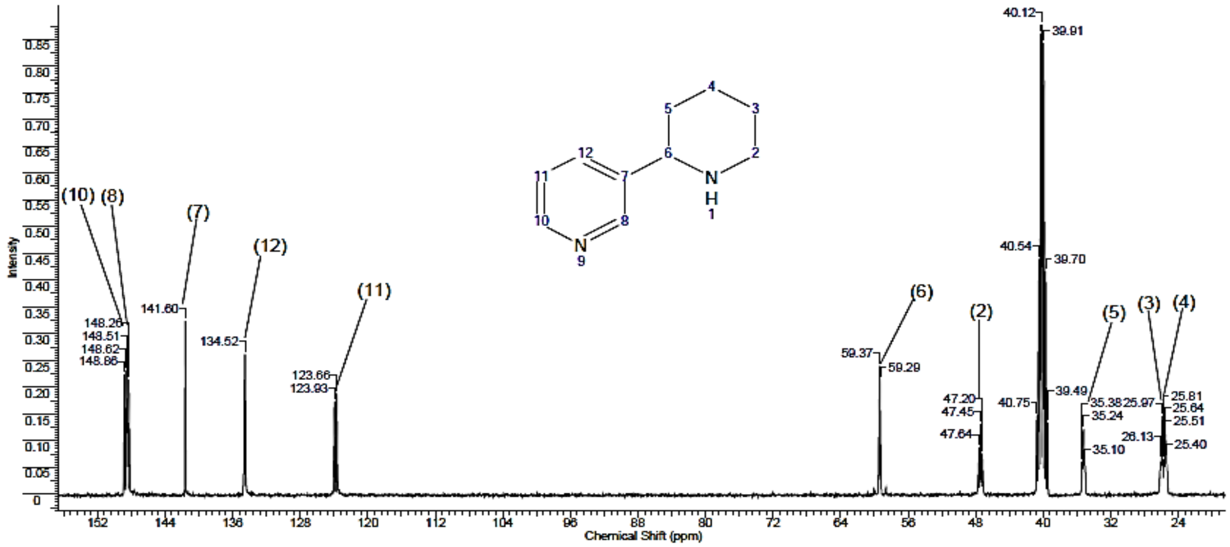

No.	(ppm)	(Hz)	Height	No.	(ppm)	(Hz)	Height	No.	(ppm)	(Hz)	Height
1	25.40	2553.5	0.0701	12	39.91	4012.3	0.8428	23	123.66	12430.5	0.1885
2	25.51	2564.0	0.1126	13	40.12	4033.4	1.0000	24	123.93	12458.3	0.1738
3	25.64	2577.4	0.0656	14	40.33	4054.4	0.8610	25	134.52	13523.1	0.2612
4	25.81	2594.7	0.0647	15	40.54	4075.5	0.4392	26	134.61	13531.8	0.2608
5	25.97	2611.0	0.1457	16	40.75	4096.6	0.1464	27	141.60	14234.3	0.3237
6	26.13	2626.3	0.0647	17	47.20	4744.5	0.0759	28	148.26	14904.3	0.2150
7	35.10	3528.2	0.0599	18	47.38	4762.8	0.0957	29	148.51	14929.2	0.2150
8	35.24	3542.6	0.1220	19	47.45	4769.5	0.1307	30	148.62	14939.8	0.2320
9	35.38	3557.0	0.0612	20	47.64	4788.6	0.0872	31	148.86	14963.7	0.2239
10	39.49	3970.1	0.1349	21	59.29	5959.9	0.2138				
11	39.70	3991.2	0.4162	22	59.37	5968.5	0.2392				

^{13}H NMR spectra of anabazine (spectrometer JNM-ECA Jeol 400 operating at 400 MHz (frequency 399.78) (25°C), DMSO-d_6)

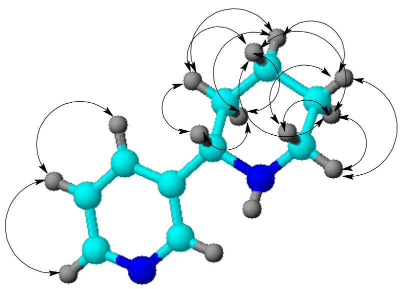

a

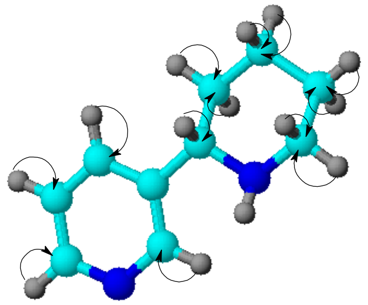

b

Correlations of COSY (^1H-^1H) and HMQC (^1H-^{13}C) in the anabazine molecule

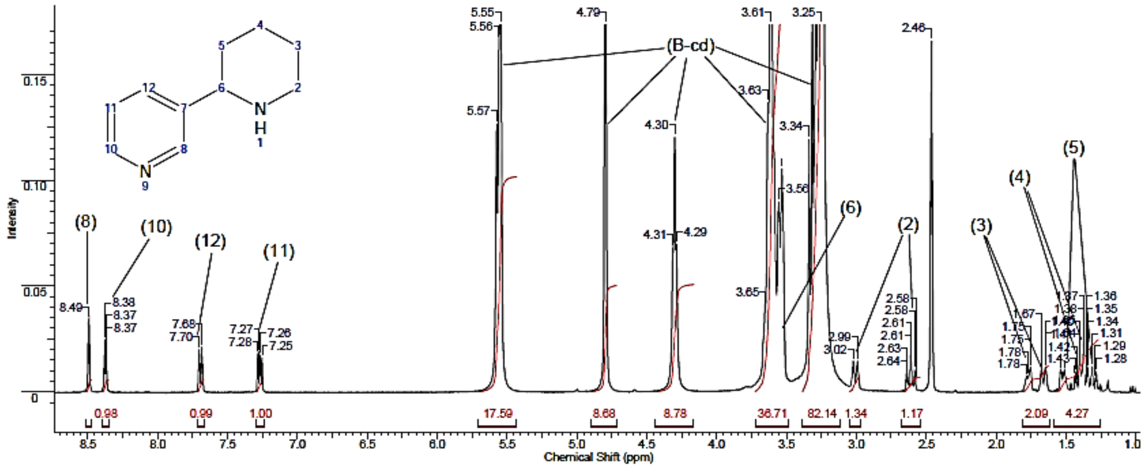

No.	(ppm)	(Hz)	Height
1	1.28	512.4	0.0097
2	1.29	516.1	0.0064
3	1.31	524.8	0.0117
4	1.32	527.5	0.0089
5	1.34	535.8	0.0114
6	1.35	539.4	0.0134
7	1.36	542.2	0.0059
8	1.37	547.2	0.0170
9	1.38	550.4	0.0229
10	1.40	559.1	0.0208
11	1.41	562.3	0.0158
12	1.42	568.7	0.0049
13	1.43	571.5	0.0095
14	1.44	574.7	0.0077
15	1.51	604.0	0.0102
16	1.54	614.1	0.0103
17	1.64	657.1	0.0114
18	1.65	659.9	0.0111
19	1.67	669.5	0.0110
20	1.75	699.7	0.0113
21	1.75	701.5	0.0115
22	1.78	710.2	0.0077
23	1.78	713.0	0.0071
24	2.46	982.7	0.1222
25	2.46	984.6	0.1658
26	2.58	1029.9	0.0062
27	2.58	1033.1	0.0105
28	2.61	1041.8	0.0138
29	2.61	1044.5	0.0185
30	2.63	1053.3	0.0086
31	2.64	1056.0	0.0088
32	2.99	1196.1	0.0142
33	3.02	1208.0	0.0143
34	3.25	1299.2	1.0000
35	3.29	1316.1	0.1873
36	3.31	1325.3	0.1839
37	3.34	1334.4	0.1196
38	3.52	1408.2	0.0836
39	3.53	1412.3	0.1055
40	3.56	1421.9	0.0900
41	3.61	1443.4	0.2721
42	3.63	1452.1	0.1368
43	3.65	1459.9	0.0412
44	4.29	1714.1	0.0697
45	4.30	1719.1	0.1204
46	4.31	1724.1	0.0681
47	4.79	1915.1	0.1964
48	4.80	1918.8	0.2031
49	5.55	2217.8	0.2118
50	5.56	2221.5	0.1669
51	5.57	2228.3	0.1267
52	7.25	2898.8	0.0160
53	7.26	2903.8	0.0173
54	7.27	2906.6	0.0188
55	7.28	2911.1	0.0178
56	7.68	3071.4	0.0212
57	7.70	3079.2	0.0201
58	8.37	3344.4	0.0242
59	8.37	3346.2	0.0254
60	8.38	3349.4	0.0245
61	8.49	3394.3	0.0340
62	8.50	3396.6	0.0326

No.	(ppm)	Value	Absolute Value
1	[1.26 .. 1.59]	4.275	7.43530e+0
2	[1.62 .. 1.81]	2.095	3.64347e+0
3	[2.54 .. 2.67]	1.172	2.03895e+0
4	[2.97 .. 3.05]	1.337	2.32604e+0
5	[3.11 .. 3.38]	82.142	1.42866e+2
6	[3.49 .. 3.72]	36.712	6.38520e+1
7	[4.16 .. 4.44]	8.780	1.52715e+1
8	[4.71 .. 4.90]	8.679	1.50954e+1
9	[5.43 .. 5.70]	17.593	3.05985e+1
10	[7.24 .. 7.30]	0.996	1.73160e+0
11	[7.67 .. 7.72]	0.993	1.72692e+0
12	[8.35 .. 8.40]	0.979	1.70303e+0
13	[8.47 .. 8.51]	0.976	1.69838e+0

^1H NMR spectra of anabazine inclusion complex with β-CD (spectrometer JNM-ECA Jeol 400 operating at 400 MHz (frequency 399.78) (25°C), DMSO-d_6)

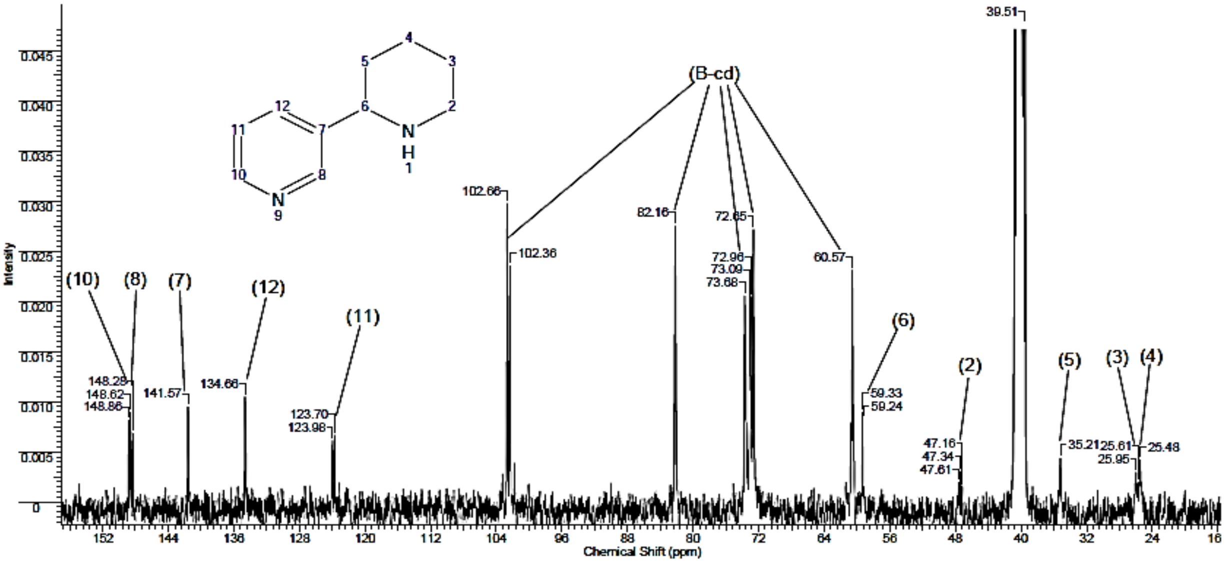

No.	(ppm)	(Hz)	Height	No.	(ppm)	(Hz)	Height	No.	(ppm)	(Hz)	Height
1	25.48	2561.2	0.0042	12	47.16	4740.7	0.0015	23	102.36	10290.2	0.0236
2	25.61	2574.6	0.0020	13	47.34	4758.9	0.0030	24	102.66	10319.9	0.0297
3	25.95	2609.1	0.0030	14	47.61	4785.8	0.0020	25	123.70	12435.3	0.0066
4	35.21	3539.7	0.0044	15	59.24	5955.1	0.0083	26	123.98	12463.1	0.0062
5	39.51	3972.0	0.1422	16	59.33	5963.7	0.0090	27	134.58	13528.9	0.0100
6	39.72	3993.1	0.4289	17	60.57	6089.3	0.0231	28	134.66	13536.6	0.0105
7	39.93	4014.2	0.8536	18	72.65	7303.6	0.0272	29	141.57	14231.4	0.0095
8	40.14	4035.3	1.0000	19	72.96	7334.3	0.0205	30	148.28	14906.2	0.0069
9	40.35	4056.4	0.8504	20	73.09	7347.7	0.0166	31	148.54	14932.1	0.0076
10	40.56	4077.4	0.4259	21	73.68	7407.2	0.0206	32	148.62	14939.8	0.0089
11	40.77	4098.5	0.1410	22	82.16	8259.2	0.0275	33	148.86	14963.7	0.0082

^{13}C NMR spectra of anabazine inclusion complex with β-CD (spectrometer JNM-ECA Jeol 400 operating at 400 MHz (frequency 399.78) (25°C), DMSO-d_6)

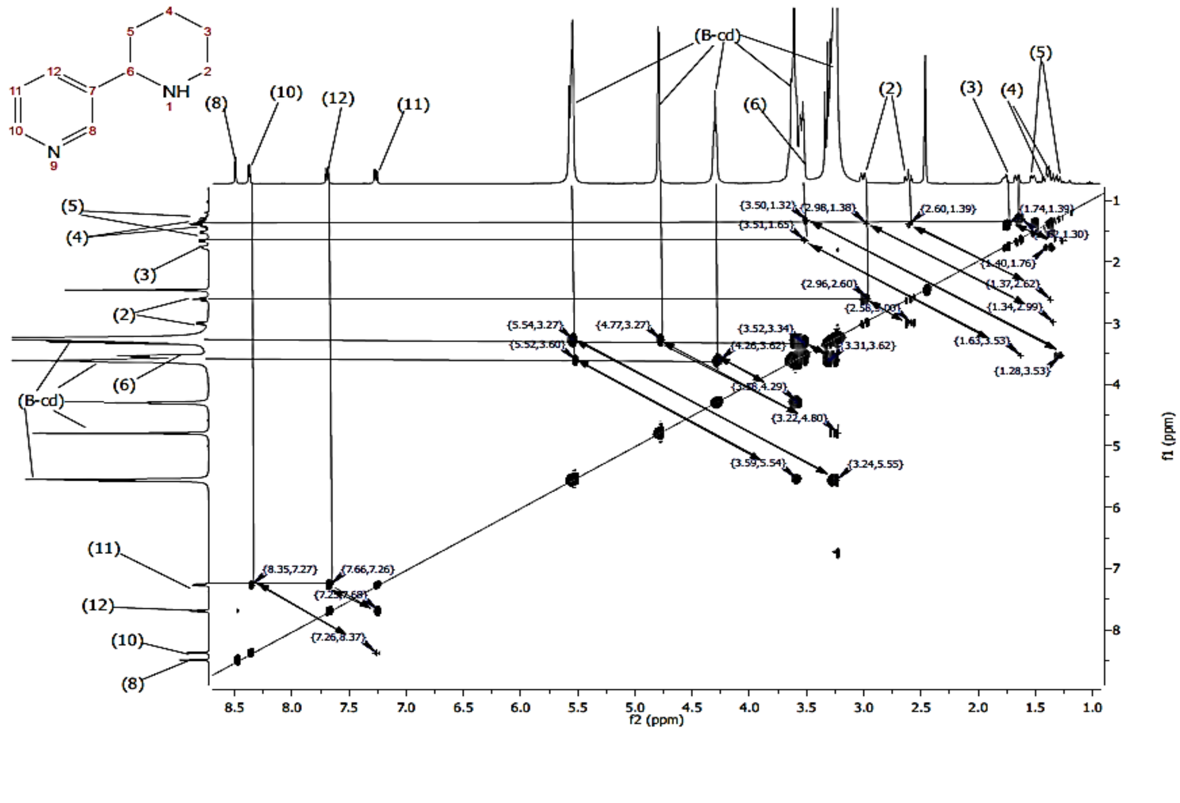

COSY (^1H-^1H) spectra of anabazine inclusion complex with β-CD

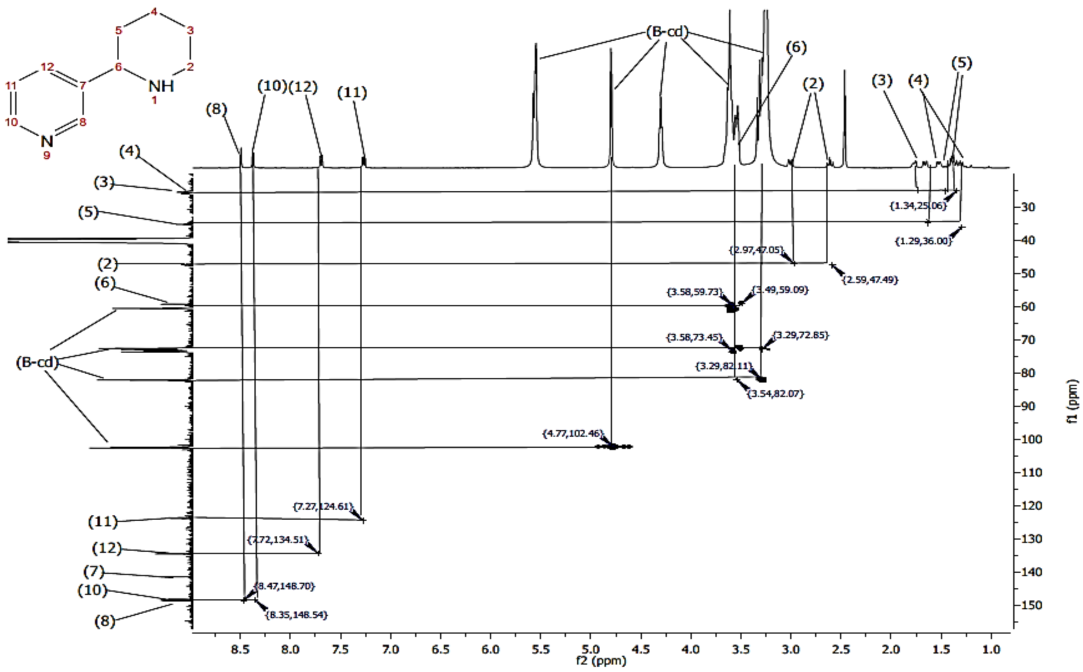

HMQC (^{1}H-^{13}C) spectra of anabazine inclusion complex with β-CD

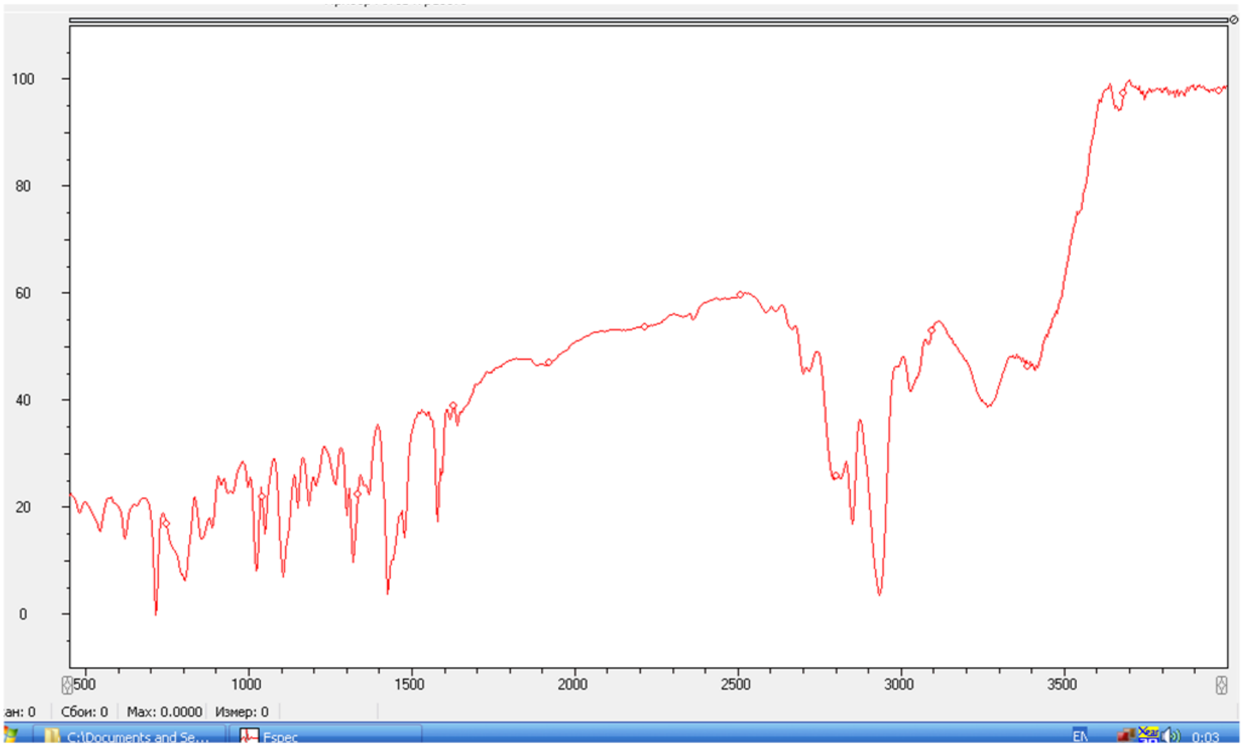

IR spectrum of anabazine alkaloid

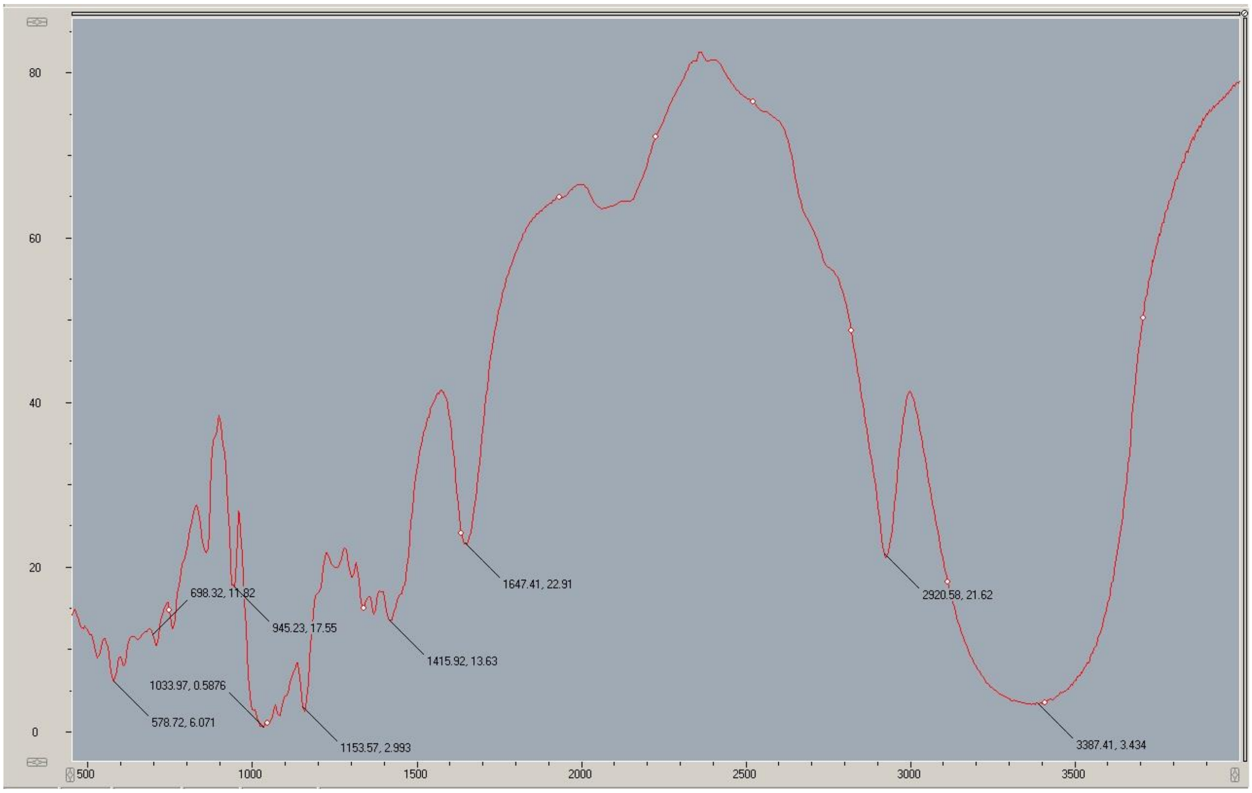

IR spectrum of beta-cyclodextrin

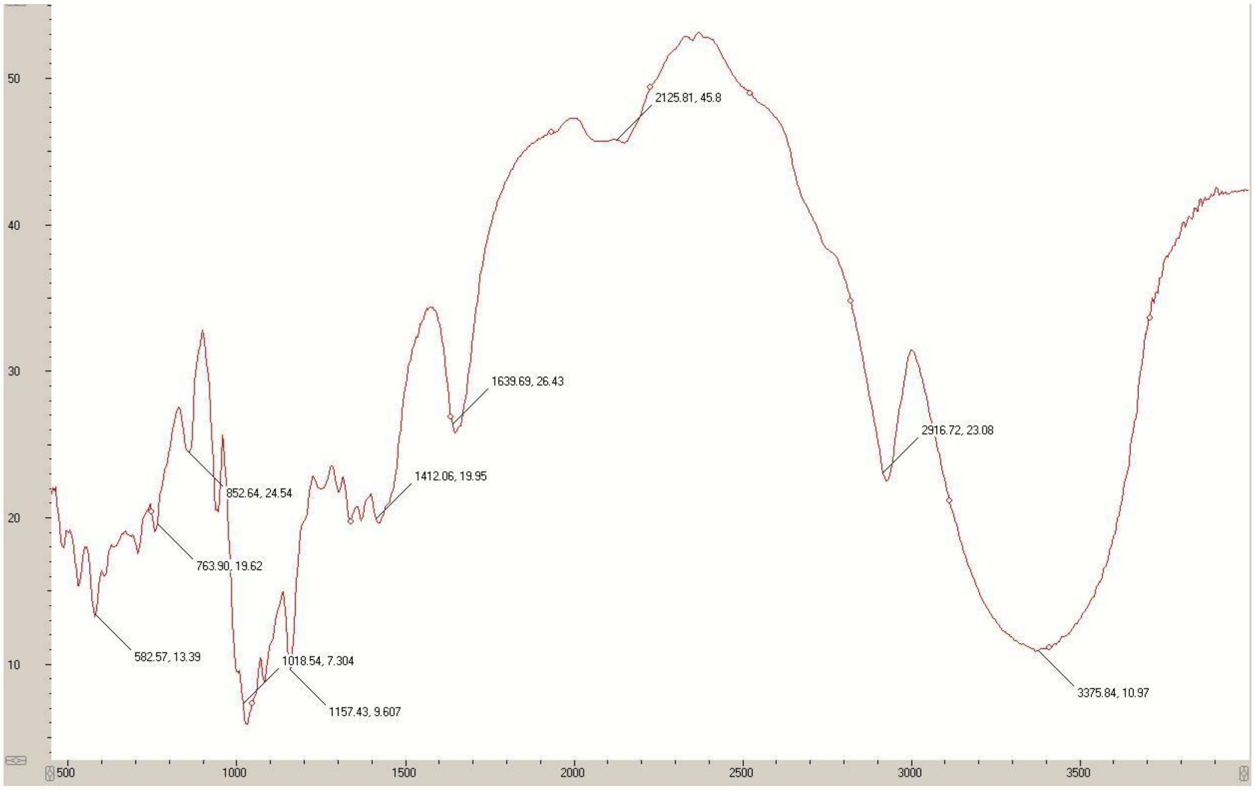

IR spectrum of the Anabazine-beta-cyclodextrin inclusion complex
